# IDO1 plays a tumor-promoting role via MDM2-mediated suppression of the p53 pathway in diffuse large B-cell lymphoma

**DOI:** 10.1038/s41419-022-05021-2

**Published:** 2022-06-27

**Authors:** Chengtao Sun, Mengzhen Li, Lian Zhang, Feifei Sun, Huimou Chen, Yanjie Xu, Yingxia Lan, Li Zhang, Suying Lu, Jia Zhu, Junting Huang, Juan Wang, Yang Hu, Yanfen Feng, Yizhuo Zhang

**Affiliations:** 1 Sun Yat-sen University Cancer Center; State Key Laboratory of Oncology in South China, Collaborative Innovation Center for Cancer Medicine, Guangzhou, Guangdong Province China; 2 Department of Pediatric Oncology, Sun Yat-sen University Cancer Center, Guangzhou, Guangdong Province China; 3 Department of Oncology, The First Affiliated Hospital of Shandong First Medical University & Shandong Provincial Qianfoshan Hospital, Shandong Key Laboratory of Rheumatic Disease and Translational Medicine, Shandong Lung Cancer Institute, Jinan, Shandong Province China; 4 Department of Pathology, Sun Yat-sen University Cancer Center, Guangzhou, Guangdong Province China

**Keywords:** Diseases, Pathogenesis

## Abstract

With the intensive therapeutic strategies, diffuse large B-cell lymphoma (DLBCL) is still a fatal disease due to its progressive characteristics. Indoleamine 2,3-dioxygenase 1 (IDO1) is a key regulator that catalyzes the commitment step of the kynurenine pathway in the immune system, its aberrant activation may contribute to malignant cell escape eradication. However, the role of IDO1 in DLBCL progression remains elusive. Our study showed IDO1 expression was upregulated in DLBCL and was associated with a poor prognosis and low overall survival. Inhibition of IDO1 suppressed DLBCL cell proliferation in vitro and impeded xenograft tumorigenesis in vivo. RNA-seq analyses revealed MDM2 was downregulated while TP53 was upregulated in IDO1 inhibition OCI-Ly10 cells. Mechanistically, IDO1 inhibition decreased the expression of MDM2, a major negative regulator of p53, and restored p53 expression in OCI-Ly3 and OCI-Ly10 cells, resulting in cell cycle arrest and apoptosis. IDO1 inhibition induced cell apoptosis coupled with PUMA and BAX upregulation, as well as BCL2 and BCL-XL downregulation. In addition, p21, a p53 transcriptional target, was upregulated in cell cycle arrest. Taken together, this study revealed IDO1 is essential for the proliferation of DLBCL cells and may be a potential therapeutic target for the treatment of DLBCL.

## Introduction

DLBCL is the most common subtype of non-Hodgkin lymphoma, accounting for more than 30% of all cases [1, 2]. DLBCL is a clinically and molecularly heterogeneous disease. With the development of the diagnosis and treatment of DLBCL, the overall survival (OS) rate is only approximately 60% over 5 years for all patients [3, 4]. Therefore, deep insight into the underlying mechanisms of DLBCL tumorigenesis is essential to identify novel biomarkers and develop promising therapies.

Recent studies found that IDO1 is upregulated in various cancers, including DLBCL [5]. Ninomiya et al. demonstrated that in DLBCL patients, 32% of cases have positive IDO expression. They also found that IDO-positive DLBCL patients have lower complete remission (CR) rates and 3-year OS rates than IDO-negative DLBCL patients [6]. Similarly, serum L-kynurenine, derived from tryptophan via IDO catalysis, is an independent prognostic factor for OS in DLBCL patients treated with the R-CHOP regimen. Furthermore, a higher serum L-kynurenine level is associated with a poor outcome [7]. However, Nam et al. found that an increased number of tumor-infiltrating IDO1-positive cells is an independent favorable prognostic factor in DLBCL treated with conventional chemotherapy [8]. These results indicated that the correlation between IDO1 overexpression and outcomes in DLBCL is inconsistent. Although some studies have shown that IDO1 is overexpressed in DLBCL [6–9], the role and mechanism of IDO1 activity in the tumor growth of DLBCL remain unclear.

IDO1, the rate-limiting tryptophan catabolic enzyme, catalyzes the commitment step of the kynurenine (KYN) biosynthesis pathway [10]. KYN is crucial for KYN pathway metabolites catalyzed by IDO1 [11]. Recent studies have revealed that KYN is an endogenous agonist of the aryl hydrocarbon receptor (AhR) [12–14]. Furthermore, the AhR pathway is activated by KYN, which leads to immunosuppression by activating dendritic cells (DCs) and regulatory T cells (Tregs), suppressing the functions of effector T and natural killer (NK) cells [10]. Recently, the IDO1-KYN-AhR signaling pathway has been well studied in various types of tumors. In addition to the role of IDO1 in immunosuppression, more researchers are currently paying attention to the role and mechanism of IDO1 independent of immune tolerance in promoting tumor development. It has been demonstrated that IDO1 deficiency reduced the density of the underlying pulmonary blood vessels and improved survival in primary lung carcinoma and breast carcinoma-derived pulmonary metastasis models [15]. Decreased IDO1 expression inhibits the tumor cell proliferation of colon cancer cells and induces mitotic death and cell cycle arrest in the G2/M phase [16]. High IDO1 expression is associated with poor outcomes in patients with breast cancer, and IDO1 promotes angiogenesis in breast cancer [17].

1-L-MT, an analog of IDO’s natural substrate L-Trp, is an IDO1 inhibitor that can competitively inhibit the enzyme activity of IDO1 and reduce the production of kynurenine. In the present study, our results demonstrated that 1-L-MT inhibited OCI-Ly3 and OCI-Ly10 DLBCL cell proliferation, induced cell apoptosis, and arrested the cell cycle.

The present study aimed to assess the relationship between IDO1 expression and clinicopathological features and prognostic value in DLBCL patients. However, the role and molecular mechanism of IDO1 in DLBCL are still unclear. Here, we evaluated the role of IDO1 in human DLBCL OCI-Ly3 and OCI-Ly10 cell growth. Furthermore, based on RNA-seq analysis performed on control (ctrl) and 1-L-MT-treated OCI-Ly10 cells, we identified MDM2 and TP53 as downstream genes of IDO1 and verified them by Quantitative reverse transcription PCR (RT-qPCR) and western blotting (WB). Collectively, we demonstrated that decreased IDO1 activity could activate the p53 pathway by suppressing MDM2 expression and inhibit DLBCL cell growth by inducing the p53 apoptotic pathway and cell cycle arrest.

## Materials and methods

### Data source

GSE56315 [18] and GSE12195 [19, 20] were downloaded from NCBI GEO databases. The microarray data of GSE56315 and GSE12195 were based on GPL570 (Affymetrix Human Genome U133 Plus 2.0 Array). GSE56315 contains the gene expression data of 89 DLBCL tissues and 33 human healthy tonsils as normal tissues. The subtypes of DLBCL in GSE56315 contained 44 germinal center B-cell (GCB), 40 activated B-cell (ABC), and 5 unclassified. GSE12195 contains the gene expression data of 73 DLBCL tissues and 10 normal tissues. The normal tissues in GSE12195 contained five germinal center centroblasts and five germinal center centrocytes from human tonsils. GSE12195 contained 73 DLBCL tissues, which did not provide information on pathological subtypes. Level 3 RNA-seq data were downloaded from the TCGA database (https://tcga-data.nci.nih.gov/tcga/), which contains 47 DLBCL patients. The limma R [21] and edge R [22] packages were used to screen differentially expressed genes (DEGs) between DLBCL samples and noncancerous tissues in the GEO and TCGA datasets, respectively. Adjusted *P* < 0.01 and | log2 FC | > 1 were used as the cutoff criteria for DEG identification. FunRich software was used to generate a Venn diagram [23].

### Clinical samples and cell lines

All DLBCL samples and clinicopathological data were obtained from the Sun Yat-sen University Cancer Center between 2006 and 2013. All patients or guardians provided written consent for the use of their data. The procedures of this study were approved by the Ethics Committee of Sun Yat-sen University Cancer Center. The wild-type *TP53* human DLBCL cell lines OCI-Ly3 and OCI-Ly10 and mutated-type *TP53* human DLBCL cells SU-DHL-6 and SU-DHL-10 were purchased from the Type Culture Collection of the Chinese Academy of Sciences (Shanghai, China). OCI-Ly3, OCI-Ly10, SU-DHL-6, and SU-DHL-10 cell lines were cultured in RPMI 1640 medium (Gibco, Thermo Fisher Scientific, USA) containing 10% fetal bovine serum (FBS, HyClone, USA) and 1% penicillin-streptomycin solution (Gibco, 15140-122, Life Technologies, USA). All cells were cultured at 37 °C in a humidified atmosphere containing 5% CO_2_. Peripheral blood mononuclear cells (PBMCs) and normal tonsil tissues were used as a control in this study.

### Immunofluorescence staining and imaging

OCI-Ly3 and OCI-Ly10 cell suspensions were fixed using 4% paraformaldehyde overnight. The next day, the DLBCL cells were permeabilized in 0.1% Triton X-100 for 5 mins and blocked in 5% BSA at room temperature for 1 hour. The cells were then incubated with a primary anti-IDO1 (ab156787, Abcam) or anti-p53 (DO-1, Santa Cruz) antibody for 1 hour and washed three times with PBS. DLBCL cells were incubated with Alexa Fluor 488-conjugated secondary antibodies (Beyotime, Shanghai, China) for 1 hour in the dark, and DAPI (Beyotime, Shanghai, China) was applied for 5 mins to counterstain the cell nuclei. The cells were centrifuged and resuspended in glass-bottom cell culture dishes (NEST, Wuxi NEST Biotechnology Co., Ltd., China). Images were acquired using a confocal microscope (LSM880, Carl Zeiss, Oberkochen, Germany). This study used ImageJ to measure the mean fluorescence intensity.

### Immunohistochemistry (IHC) staining and imaging

Immunohistochemical staining for IDO1, MDM2, and p53 was performed in the present study. The paraffin-embedded sections of DLBCL were incubated with a primary anti-IDO1 antibody (ab55305, Abcam) at 4 °C overnight. Meanwhile, paraffin-embedded sections of tumor tissues from mice bearing OCI-Ly3 or OCI-Ly10 xenografts were incubated with a primary anti-MDM2 (556353, BD Pharmingen) or anti-p53 (DO-1, Santa Cruz) at 4 °C overnight. The next morning, the sections were incubated with goat anti-mouse secondary antibodies (Zhongshan Goldbridge Biotechnology CO., Ltd, Beijing, China) at 37 °C for 1 hour. All images were observed under a positive fluorescence microscope (Olympus IX73, Tokyo, Japan).

### Cell counting kit (CCK-8) assay

DLBCL cells were plated into 96-well plates at a density of 5×10^3^ cells/well in a final volume of 200 μL medium. The cells were then stimulated with 1-L-MT (Sigma-Aldrich, USA), an IDO1 inhibitor, at different concentrations and incubated at 37 °C for 48 hours. Following incubation, 10% CCK-8 (APExBIO, K1018, Houston, USA) was added per well. The absorbance was measured at 450 nm using a microplate reader (Tecan, M200 PRO, Switzerland).

### EdU cell proliferation assay

First, OCI-Ly3 and OCI-Ly10 cells were treated with 1-L-MT for 48 hours. DLBCL cells were then incubated with 50 μM EdU (RiboBio, C10310-3, Guangzhou, China) for 2 hours at 37 °C. After fixation with 4% paraformaldehyde and washing with PBS, the cells were stained with 1× Apollo for 30 mins in the dark. Then, 1× Hoechst 33342 was used to stain cell nuclei. Finally, the EdU-stained cells were centrifuged and resuspended in glass-bottom cell culture dishes, and images were acquired by a confocal microscope (LSM880, Carl Zeiss, Oberkochen, Germany).

### Enzyme-linked immunosorbent assay (ELISA)

The human kynurenine ELISA Kit was purchased from SenBeiJia Biological Technology Co., Ltd. (SBJ-H2393, Nanjing, China). OCI-Ly3 and OCI-Ly10 cells were treated with 1-L-MT (5 mM) and Epacadostat (INCB024360) (50 uM) for 24 h. KYN was examined by Elisa according to the manufacturer’s protocol.

### Xenograft tumorigenesis in NOD/SCID mice

The animal experiments were conducted according to the Guide for the Care and Use of Laboratory Animals and approved by the Ethics Committee of the Sun Yat-sen University Cancer Center. A total of 5 × 10^6^ live OCI-Ly10 cells or OCI-Ly3 cells were suspended in 100 μL of PBS and inoculated subcutaneously into four-week-old NOD/SCID female mice (Vital River Laboratory Animal Technology, Beijing, China). Nine days later, 10 mice were randomly divided into 2 groups. The 1-L-MT group mice were given a drinking water supplement with 1-L-MT (400 mg/kg). The control mice were fed with water without 1-L-MT. The tumor size was monitored 2-3 times per week with a Vernier caliper. The tumor volume was calculated as (length × width^2^)/2. Once the tumor length of any mouse was greater than 20 mm, the remaining mice were euthanized.

### RNA-seq analysis

Biological triplicate RNA-seq analysis was performed on 6 independent RNA samples of IDO1 inhibition OCI-Ly10 cells (3 samples) and ctrl OCI-Ly10 cells (3 samples). Total RNA was extracted using TRIzol (Ambion, Life Technologies, USA) according to the manufacturer’s protocol. RNA-seq libraries were constructed using the Wuxi in-house mRNA library construction kit and then sequenced by NovaSeq 6000 (WuXi NextCODE, Shanghai, China) in PE150 mode. Raw reads were trimmed by Skewer (v0.2.2) [24] to remove adapter sequences and then aligned against the reference human (Homo sapiens) genome (version hg19) by STAR (v2.4.2a) [25]. RSEM (V1.2.29) [26] were used to perform expression abundance quantification based on the uniquely mapped reads. Edge R was used to identify DEGs, and adjusted *P* < 0.01 and | log2 fold change (1-L-MT vs. ctrl) | ≥ 0.5 were set as the cutoff criteria. Gene ontology (GO) and Kyoto Encyclopedia of Genes and Genomes (KEGG) enrichments were analyzed using the Database for Annotation, Visualization, and Integrated Discovery (DAVID, version 6.7, https://david.ncifcrf.gov/) Functional Annotation Tools. The heatmap was generated using FunRich software [23]. Gene set enrichment analysis (GSEA) was performed with GSEA 4.1.0 software.

### Reverse transcription-quantitative (RT-q) PCR

Total RNA was extracted by using TRIzol (Ambion, Life Technologies, USA) according to the manufacturer’s protocol. The concentration and purity of the RNA were analyzed using a NanoDrop 2000 (Thermo Fisher Scientific, Waltham, MA, USA). Then, total RNA was reverse transcribed to cDNA, and subsequently, qPCR was performed on the Bio-Rad CFX96 system with Power SYBR Green qPCR Mix (Dongsheng Biotech Co., Ltd, P2091, Guangzhou, China). The 2^-ΔΔCq^ method was utilized for the quantification of gene expression, with β-actin as an endogenous control. Primers for MDM2 (forward, 5’-GGCGTGCCAAGCTTCTCTGTG-3’; reverse, 5’-ACCTGAGTCCGATGATTCCTGCTG-3’), TP53 (forward, 5’-ACCGGCGCACAGAGGAAGAG-3’; reverse, 5’-GCCTCATTCAGCTCTCGGAACATC-3’), P21 (forward, 5’-GATGGAACTTCGACTTTGTCAC-3’; reverse, 5’-GTCCACATGGTCTTCCTCTG-3’), PUMA (forward, 5’-GCCAGATTTGTGAGACAAGAGG-3’; reverse, 5’-CAGGCACCTAATTGGGCTC-3’), BAX (forward, 5’-CCCGAGAGGTCTTTTTCCGAG-3’; reverse, 5’-CCAGCCCATGATGGTTCTGAT-3’) and β-actin (forward, 5’-CCTGGCACCCAGCACAAT-3”; reverse, 5’-GGGCCGGACTCGTCATAC-3’) were obtained from Sangon Biotech Co., Ltd. (Shanghai, China).

### Western blotting analysis

The cells from each group were collected, and lysates were generated using the KeyGEN kit (KGP2100, Nanjing, China). The protein concentration was assessed by the BCA method (Thermo Fisher Scientific, Waltham, USA). Next, equal amounts of proteins were analyzed by WB using the following antibodies: anti-MDM2 (556353, BD Pharmingen), anti-p53 (DO-1, Santa Cruz), anti-IDO1 (ab55305, Abcam), anti-p21 (ab109199, Abcam), anti-BCL2 (ab182858, Abcam), anti-BCL-XL (ab32370, Abcam), anti-PUMA (ab33906, Abcam), anti-BAX (ab32503, Abcam) and anti-β-actin (P60709, Cell Signaling Technology). The signals were detected using an ECL chemiluminescence detection system (Bio-Rad, USA). The unprocessed WB images were shown in Supplementary Fig. S4.

### Flow cytometry analysis

An Annexin V-Alexa Fluor 647/7-AAD Kit (4 A Biotech Co., Ltd., Beijing, China) was used to evaluate cell apoptosis. DLBCL cells were centrifuged and washed twice with cold PBS. Cells were resuspended in 1× binding buffer at a cell density of 1 × 10^6^ cells/ml. Five microliters of Annexin V/Alexa Fluor 647 were coincubated with 100 µl of the cell suspensions for 5 mins at room temperature in the dark. Then, 10 µl 7-AAD (20 µg/ml) and 400 µl PBS were added to the cells, and the stained cells were analyzed by flow cytometry (SP6800, Sony, Japan) within 1 hour.

As previously described [27], DLBCL cells were fixed with precooled 70% alcohol overnight and stained with PI/RNase Staining Buffer (Beyotime, Shanghai, China) at 37 °C for 30 mins in the dark. The cell cycle distribution was determined using ModFit LT software (BD, Topsham, ME, USA). Cell apoptosis was analyzed by FlowJo (Tree Star, Inc., Ashland, OR).

### Statistical analysis

A two-sided student’s t-test was used to detect the statistical significance between two groups. The relationship between IDO1 expression and clinicopathological characteristics in DLBCL patients was analyzed using the two-sided χ^2^ test. Kaplan-Meier survival curves were used to compare survival rates between different groups. SPSS 20.0 software (SPSS, Chicago, IL, USA) was used to perform statistical analyses. A *P*-value < 0.05 was considered statistically significant. The receiver operating characteristic (ROC) curves and precision recall curves (PRC) analyses were performed by using R package *modEvA*. The confusion matrix was obtained by utilizing R package *caret*.

## Results

### IDO1 was overexpressed in DLBCL based on GEO and TCGA datasets

**Fig. 1:**
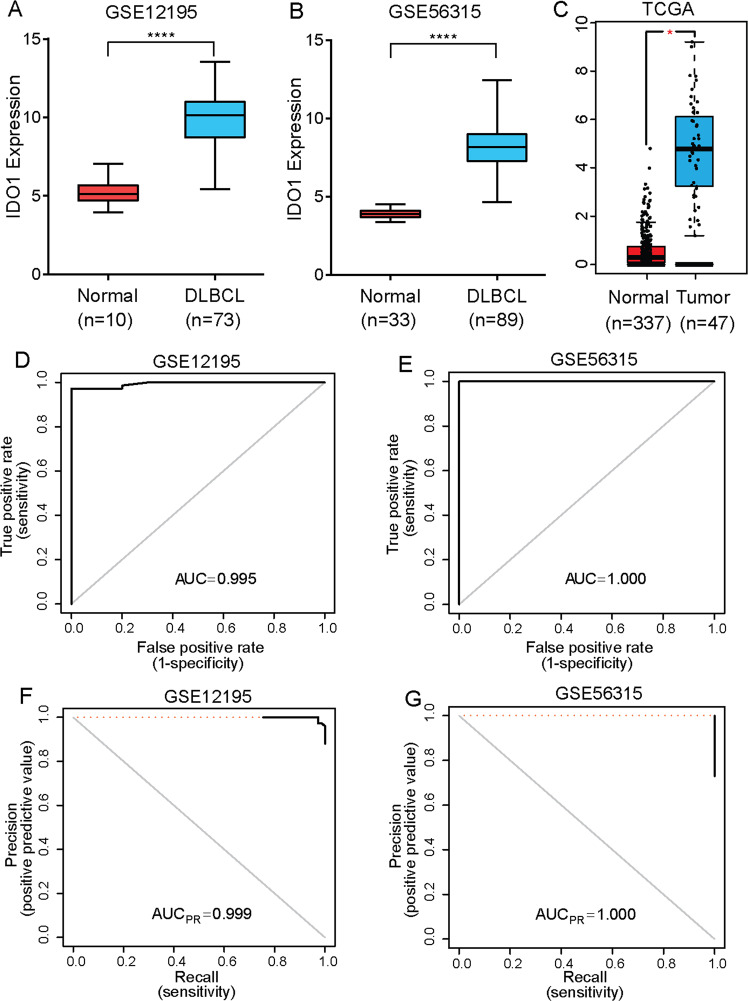
DLBCL datasets revealed that IDO1 was overexpressed in DLBCL and could be a predictive marker for DLBCL. **A**–**C** IDO1 expression in DLBCL and normal tissues from GSE12195 (**A**), GSE56315 (**B**), and TCGA DLBCL (**C**). **D**, **E** Receiver operating characteristic (ROC) curves calculated the sensitivity and specificity of the IDO1 expression level for the prediction of DLBCL in the GSE12195 (**D**) (AUC = 0.995) and GSE56315 (**E**) (AUC = 1.000) datasets. **F, G** PRC analysis calculated sensitivity and specificity of the IDO1 expression level for the prediction of DLBCL in the GSE12195 (**F**) (AUC = 0.999) and GSE56315 (**G**) (AUC = 1.000) datasets. Statistical analysis was analyzed with a two-sided student’s t-test. *P < 0.05 and ****P < 0.0001.

The bioinformatic analysis results of GSE12195 showed that when compared with normal tissues, IDO1 was elevated in 73 DLBCL tissues (*P* < 0.0001, Fig. 1A). As shown in Fig. 1B, the IDO1 expression level was significantly upregulated in 89 DLBCL tissues compared with 33 normal tissues in GSE56315 (*P* < 0.0001, Fig. 1B). Furthermore, we measured IDO1 mRNA expression in the TCGA DLBCL dataset and observed a similar expression trend (*P* < 0.05, Fig. 1C). Accordingly, we constructed the classification model to evaluate the diagnostic performance of IDO1 in distinguishing tumor from normal sample. Specifically, we applied a logistic regression, in which the expression value of IDO1 was used as the independent variable, to calculate the probability of a sample to be a tumor. We have conducted the ROC curves (Fig. 1D, E) as well as PRC analyses in the two datasets (Fig. 1F, G). The area under the ROC curve (AUC) of IDO1 in GSE12195 and GSE56315 was 0.995 and 1.000, respectively. While the area under the PRC curves was 0.999 and 1.000, respectively. Moreover, the confusion matrix has been provided in our supplemental material (Supplementary Table S1). The results above indicated that IDO1 could be a new predictive marker in DLBCL.

### IDO1 was associated with a poor outcome, and IDO1 inhibition suppressed tumorigenesis in NOD/SCID mice

**Fig. 2:**
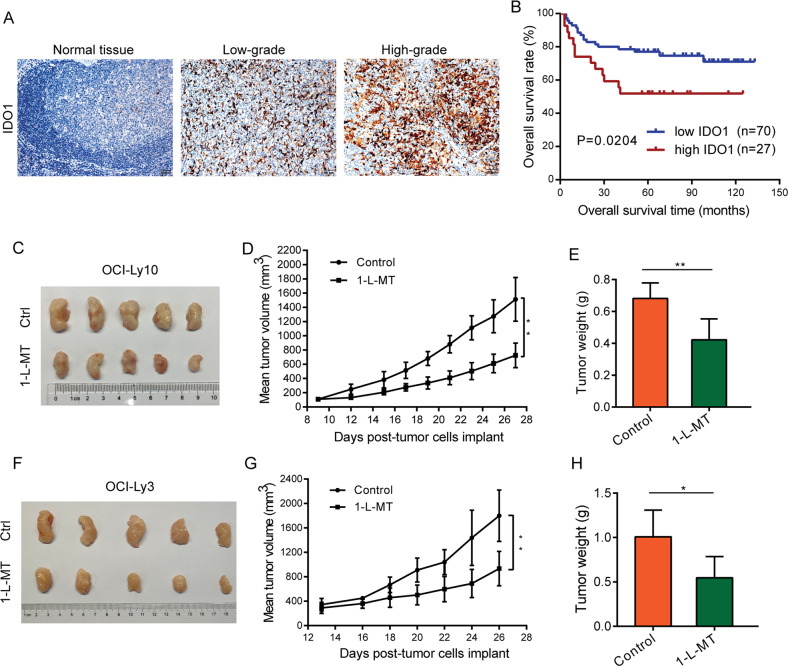
High IDO1 expression predicted poor outcome, and IDO1 inhibition suppressed tumorigenesis in NOD/SCID mice. **A** Representative IHC images of IDO1 in normal tissue and 97 DLBCL patient samples (× 200, scale bar, 50 μm). **B** Kaplan-Meier OS analysis of DLBCL patients with high IDO1 expression and low IDO1 expression levels (*P* = 0.0204, *n* = 97). **C** and **F** A total of 5×10^6^ OCI-Ly10 (**C**) or OCI-Ly3 (**F**) cells were subcutaneously injected into the posterior flank of NOD/SCID mice. Representative images of xenograft tumors treated with or without 1-L-MT are presented. **D** and **G** Tumor volume of OCI-Ly10 (**D**) or OCI-Ly3 (**G**) tumor-bearing mice demonstrated that the 1-L-MT-treated group presented a smaller size than the control group. **E** and **H** 1-L-MT-treated OCI-Ly10 (**E**) or OCI-Ly3 (**H**) tumor-bearing mice formed tumors with lower weights compared to the control group. Statistical analysis was analyzed with a two-sided student’s t-test. *P < 0.05 and **P < 0.01.

IHC staining in 97 patients enrolled in our hospital was used to confirm the elevation of IDO1 expression in DLBCL tissues (Fig. 2A, Supplementary Fig. S1). Furthermore, the correlation between IDO1 expression and clinicopathological variables was listed in Table 1. The IDO1 expression level had a close correlation with the clinical stage by Ann Arbor (χ2 = 5.033, *P* = 0.025) and IPI risk (χ2 = 5.138, *P* = 0.023). However, the association between IDO1 expression and sex, age, B symptoms, LDH, number of extranodal sites, and subtypes of DLBCL showed no significance (Table 1, *P* > 0.05). These results indicated that IDO1 expression correlated with adverse clinical features in DLBCL. We next evaluated the effects of IDO1 on overall survival in DLBCL patients. In 97 DLBCL cases, a higher IDO1 expression was significantly associated with unfavorable OS (Fig. 2B, *P* = 0.0204).

**Table 1:** Correlation between IDO1 expression and clinicopathological features of 97 DLBCL patients.

		IDO1			
Characteristics	Cases, *n*	Lower, *n*	Higher, *n*	χ^2^	*P*-value
Sex					
Male	49	39	10	2.719	0.099
Female	48	31	17		
Age					
≤60	63	48	15	1.450	0.229
>60	34	22	12		
Stage					
I–II	39	33	6	5.033	0.025
III–IV	58	37	21		
B symptoms					
No	51	39	12	0.993	0.319
Yes	46	31	15		
LDH					
Normal	59	45	14	1.264	0.261
Elevated	38	25	13		
IPI risk group					
0–2	70	55	15	5.138	0.023
3–5	27	15	12		
No. of extranodal sites					
0–1	84	61	23	0.064	0.800
≥2	13	9	4		
subtypes of DLBCL					
GCB	15	10	5	0.189	0.664
Non-GCB	30	18	12		

*DLBCL* diffuse large B-cell lymphoma, *IDO1* indoleamine 2,3-dioxygenase 1, *IPI* international prognostic index, *LDH* lactate dehydrogenase, *GCB* germinal center B-cell, *non-GCB* non-germinal center B cell. Statistical analysis was analyzed with a two-sided χ^2^ test.

In addition, to investigate the role of IDO1 in suppressing tumorigenicity in vivo, we used the NOD/SCID mouse xenograft model bearing OCI-Ly10 cells treated with 1-L-MT. As illustrated in Fig. 2C, D, compared with the control groups, 1-L-MT significantly inhibited the tumor growth rate and reduced the tumor size (*P* < 0.01, Fig. 2D). Furthermore, the average tumor weight in the 1-L-MT groups was lighter than that of the controls (*P* < 0.01, Fig. 2E). Besides, we also used another cell line OCI-Ly3 to perform animal experiments and got similar results with the OCI-Ly-10 cell line (Fig. 2F–H). These results indicated that 1-L-MT could inhibit DLBCL tumor growth in vivo.

### IDO1 inhibitor suppressed cell growth and induced cell apoptosis and G2/M arrest in DLBCL cells

**Fig. 3:**
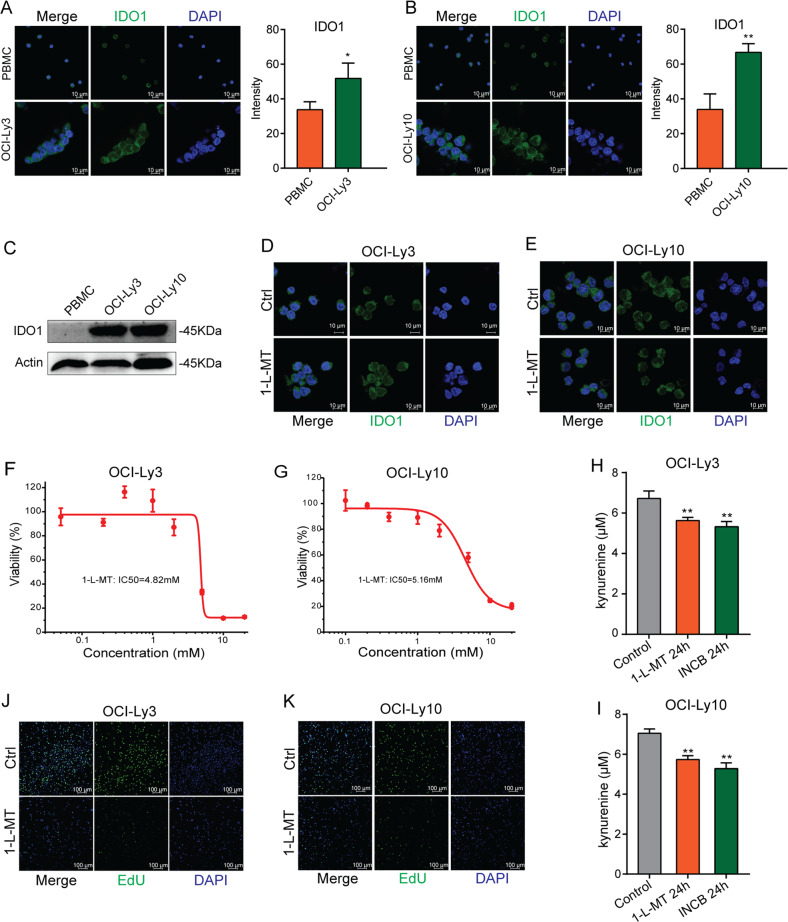
IDO1 inhibitor suppressed DLBCL cell growth. **A**, **B** Immunofluorescence staining for IDO1 expression in PBMCs, OCI-Ly3 cells, and OCI-Ly10 cells (× 630, scale bar, 10 μm). **C** WB analysis of IDO1 protein expression in OCI-Ly3, OCI-Ly10, and PBMCs. **D, E** 1-L-MT competitively inhibited the activity of IDO1 and did not affect the protein expression level of IDO1 by immunofluorescence assay (× 630; scale bar, 10 μm). **F, G** Cell viability was determined using CCK-8 after OCI-Ly3 and OCI-Ly10 cells were treated with various concentrations of 1-L-MT for 48 h. **H,I** OCI-Ly3 and OCI-Ly10 cells were stimulated with 5 mM 1-L-MT or 50 μM INCB for 24 h, and then ELISA was used to determine the KYN acid secretion. **J,K** EdU staining of 5 mM 1-L-MT-treated and control OCI-Ly3/OCI-Ly10 cells (×100; scale bar, 100 μm). Statistical analysis was analyzed with a two-sided student’s t-test. *P < 0.05 and **P < 0.01.

Immunofluorescence staining showed that the fluorescence intensity of OCI-Ly3 and OCI-Ly10 cells tends to be stronger than that of PBMC without taking into account the size of PBMC or DLBCL cells (Fig. 3A, B). Furthermore, the WB was performed and the results demonstrated that IDO1 protein levels were elevated in OCI-Ly3 and OCI-Ly10 cells compared with PBMCs (Fig. 3C). Taken together, these findings indicated that IDO1 was highly expressed in DLBCL cells.

The above findings indicated that IDO1 might play a potential role in promoting DLBCL. To investigate the possible role of IDO1 in DLBCL cells, an IDO1 inhibitor, 1-L-MT was used. As a tryptophan analog, 1-L-MT can competitively inhibit the activity of IDO1 and does not affect the protein expression level of IDO1 (Fig. 3D, E). As shown in Fig. 3F, G, by using the CCK-8 assay, we found that inhibition of IDO1 by 1-L-MT significantly suppressed the proliferation of OCI-Ly3 and OCI-Ly10 cells. Then, EdU staining showed that the percentage of EdU-positive cells was significantly decreased in OCI-Ly3 and OCI-Ly10 cells treated with 5 mM 1-L-MT for 48 h compared to control cells (Fig. 3J, K). Besides, IDO1 catabolizes tryptophan through kynurenine pathway. IDO1 inhibitors can inhibit the expression of KYN by inhibiting IDO1 enzyme activity. We treated OCI-Ly3 and OCI-Ly10 cells with 1-L-MT and INCB (another IDO1 inhibitor) for 24 h, and measured the KYN concentration in the supernatant of the culture medium by using the ELISA method. The results showed that after treatment with two different IDO1 inhibitors, the KYN concentration was significantly decreased (Fig. 3H, I).

**Fig. 4:**
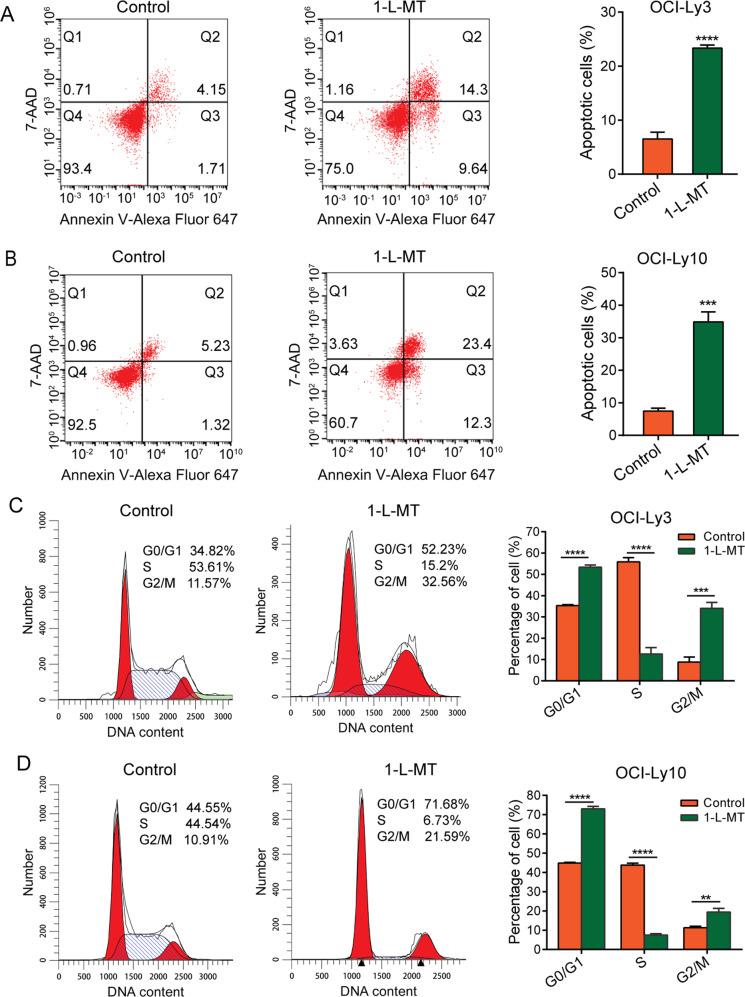
IDO1 inhibition induced cell apoptosis and G2/M arrest in DLBCL cells. **A, B** At 48 h after stimulation with 5 mM 1-L-MT, annexin V-Alexa Fluor 647 and 7-AAD staining was performed. Then, the rate of apoptotic cells was analyzed by flow cytometry in OCI-Ly3 and OCI-Ly10 cells. **C, D** OCI-Ly3 and OCI-Ly10 cells were treated with 5 mM 1-L-MT for 24 h. Then, the cell cycle distribution was analyzed using flow cytometry. Statistical analysis was analyzed with a two-sided student’s t-test. **P < 0.01, ***P < 0.001 and ****P < 0.0001.

To investigate whether IDO1 was associated with apoptosis, OCI-Ly3 and OCI-Ly10 cells were stimulated with 5 mM 1-L-MT for 48 h. As illustrated in Fig. 4A, B, the percentage of apoptotic cells was remarkably increased in 1-L-MT-treated OCI-Ly3 and OCI-Ly10 cells compared with control cells. For cell cycle analysis, DLBCL cells were stimulated with 5 mM 1-L-MT for 24 h. The cell cycle distribution results indicated that 1-L-MT-treated OCI-Ly3 cells had a G2/M phase arrest (32.56% vs. 11.57%, *P* < 0.001) compared to control cells (Fig. 4C). Similarly, OCI-Ly10 cells stimulated with 5 mM 1-L-MT for 24 h also showed a G2/M arrest (21.59% vs. 10.91%, *P* < 0.01) compared to control cells (Fig. 4D). In addition, DLBCL cells treated with 1-L-MT also showed a higher number of cells in the G0/G1 phase and a reduced number of cells in the S phase (Fig. 4C and D).

### RNA-seq analysis revealed that MDM2 and TP53 were genes downstream of IDO1

**Fig. 5:**
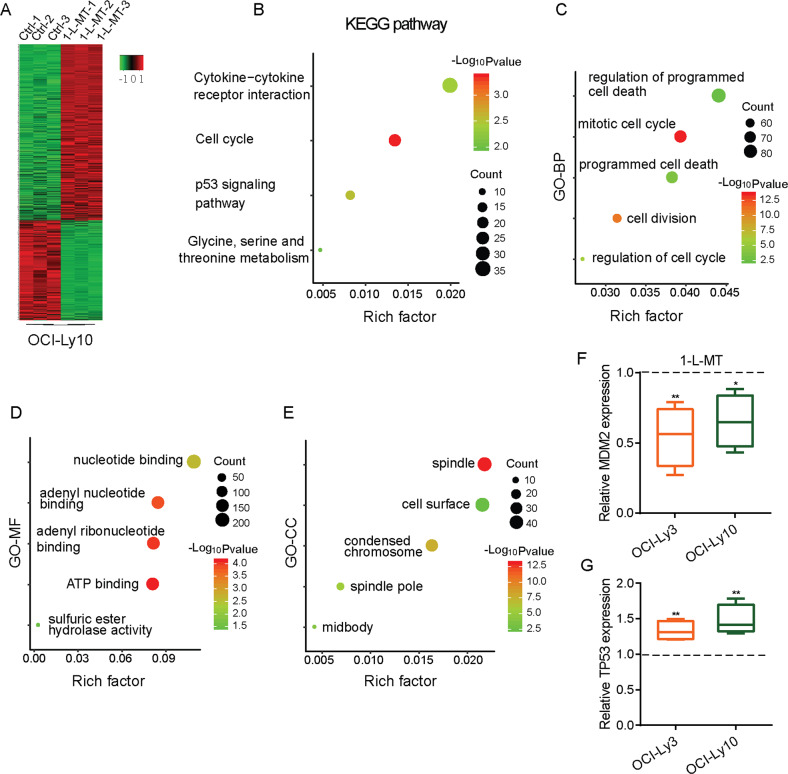
MDM2 and TP53 were genes downstream of IDO1 in DLBCL. **A** The cluster heat map is classified as DEGs in IDO1 inhibition compared with Ctrl OCI-Ly10 cells. **B**–**E** Significantly enriched KEGG (**B**) and GO (**C**–**E**) pathway terms of the DEGs in IDO1 inhibition OCI-Ly10 cells were identified. **F** RT-qPCR was used to assess the mRNA levels of MDM2 in 1-L-MT and Ctrl OCI-Ly3/OCI-Ly10 cells. **G** RT-qPCR validation of TP53 mRNA levels in 1-L-MT and Ctrl OCI-Ly3/OCI-Ly10 cells. Statistical analysis was analyzed with a two-sided student’s t-test. *P < 0.05 and **P < 0.01.

To investigate the potential genes downstream of IDO1 in DLBCL, we performed RNA-seq analysis on IDO1 inhibition and Ctrl OCI-Ly10 cells. As shown in Fig. 5A, compared with control cells, 1248 upregulated and 669 downregulated genes in 1-L-MT-treated OCI-Ly10 cells were identified (|log2 fold change (1-L-MT vs. Ctrl) | ≥ 0.5, adjusted *P* < 0.01) (Supplementary Table S2). The KEGG pathway enrichment analyses indicated that all DEGs were mainly involved in the cell cycle, p53 signaling pathway, cytokine-cytokine receptor interaction, and glycine, serine, and threonine metabolism (Fig. 5B). GO analysis of the DEGs showed involvement in biological processes (BP), molecular function (MF), and cellular component (CC). For BP, the categories included mitotic cell cycle, cell division, cell cycle regulation, programmed cell death, and regulation of programmed cell death (Fig. 5C). The MF categories were largely enriched for ATP binding, adenyl ribonucleotide binding, adenyl nucleotide binding, nucleotide binding, and sulfuric ester hydrolase activity (Fig. 5D). The CC categories were mainly enriched in the spindle, condensed chromosome, spindle pole, midbody, and cell surface (Fig. 5E). Among 669 downregulated genes in IDO1 inhibition OCI-Ly10 cells, MDM2 was one of the most downregulated genes. MDM2 overexpression was observed in 43% of DLBCL patients [28] and linked with a poor 5-year OS and median disease-free survival in DLBCL with the wild-type TP53 gene [29]. The MDM2 gene is transactivated by p53, which is subsequently perturbed by MDM2-mediated ubiquitination, thereby forming a negative feedback loop [30]. Furthermore, dysregulation of MDM2-p53 loop plays a key role in the pathogenesis and prognosis of DLBCL. Thus, we chose the MDM2-p53 loop from RNA-seq analysis for further investigation. RT-qPCR validation showed that MDM2 and TP53 mRNA expression had the same expression trend as quantified by RNA-seq analysis in 1-L-MT, control OCI-Ly3, and OCI-Ly10 cells (Fig. 5F, G). Therefore, based on our findings from RNA-seq and RT-qPCR validation, we deduced that the MDM2-p53 loop were genes downstream of IDO1.

### The role of the IDO1-MDM2-p53 signaling pathway in DLBCL cells

**Fig. 6:**
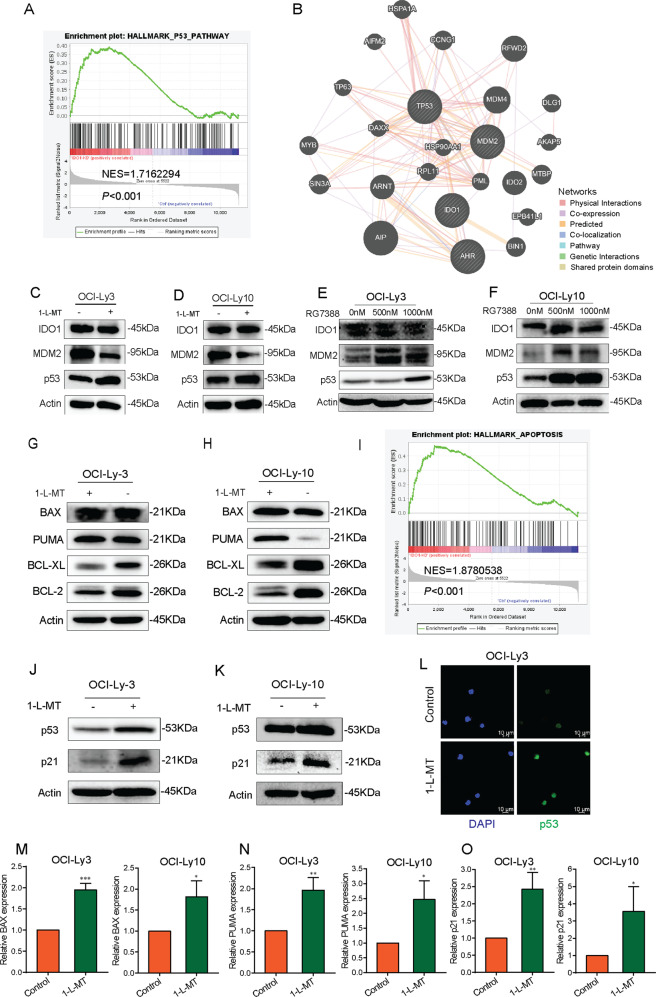
The role of the IDO1-MDM2-p53 signaling pathway in DLBCL cells. **A** GSEA showed DEGs enriched in the p53 pathway in IDO1 inhibition OCI-Ly10 cells. **B** The molecular functional network of IDO1-MDM2-p53 was constructed using GeneMANIA (http://genemania.org/). **C-D** OCI-Ly3 and OCI-Ly10 cells were treated with 5 mM 1-L-MT for 24 h. The expression levels of MDM2 and p53 were detected using WB. **E, F** OCI-Ly3 and OCI-Ly10 cells were treated with RG7388 for 24 h. The expression levels of IDO1, MDM2, and p53 were assessed using WB. **G, H** Proteins related to apoptosis, including the proapoptotic proteins BAX and PUMA and the antiapoptotic proteins BCL-2 and BCL-XL, were assessed by WB. **I** GSEA showed DEGs enriched in apoptosis in OCI-Ly10 cells treated with 1-L-MT. **J, K** The protein levels of cell cycle markers, including p21 and p53, were assessed by WB. The results showed increased expression of p21 coupled with elevated p53 levels in OCI-Ly3 (**J**) and OCI-Ly10 (**K**) cells. **L** Immunofluorescence staining confirmed the increased levels of p53 in OCI-Ly3 cells after 24 h of treatment with 1-L-MT. **M**–**O** The expression of BAX (**M**), PUMA (**N**), and P21 (**O**) was assessed by RT-qPCR in OCI-Ly3 and OCI-Ly10 after treatment with 1-L-MT for 24 h. Statistical analysis was analyzed with a two-sided student’s t-test. *P < 0.05, **P < 0.01 and ***P < 0.001.

GSEA enrichment showed DEGs involved in the p53 pathway in OCI-Ly10 cells which were stimulated with 1-L-MT (NES = 1.7162294, *P* < 0.001, Fig. 6A). As shown in Fig. 6B, the online GeneMANIA (http://genemania.org/) tool was used to generate a molecular network [31]. Three genes, TP53, MDM2 and AhR, associated with IDO1 were selected as key fixed nodes after pathway analysis (Fig. 6B). As previously introduced, AhR, a ligand-activated helix-loop-helix transcription factor involved in regulating biological responses to planar aromatic hydrocarbons, was widely study in various of tumors [32]. MDM2 can promote tumor formation by targeting p53, for proteasomal degradation. This gene is itself transcriptionally-regulated by p53 [33]. IDO1 inhibitor significantly suppressed MDM2 and restored p53 protein expression in OCI-Ly3 and OCI-Ly10 cells treated with 1-L-MT (5 mM) for 24 h (Fig. 6C, D). IHC were used to evaluate the expression levels of MDM2 and p53 in vivo. It was disclosed that treatment with 1-L-MT remarkably reduced the expression level of MDM2 in mice bearing OCI-Ly3 and OCI-Ly10 xenografts, while expression level of p53 was increased in 1-L-MT treated group compared with control group (Supplementary Fig. S3). RG7388 is a selective second-generation MDM2 inhibitor that disrupts the MDM2-p53 interaction and activates p53 [34]. OCI-Ly3 and OCI-Ly10 cells were treated with 500 nM and 1000 nM RG7388 for 24 h. WB results showed that the protein expression of MDM2 and p53 was increased, but they did not affect the protein expression of IDO1 (Fig. 6E, F). Besides, we tested two *TP53* mutated cell lines, SU-DHL-6 and SU-DHL-10, we found that the expression of MDM2 and p53 could not be affected after treatment with 1-L-MT in these two cell lines (Supplementary Fig. S2A–F). Furthermore, there were no loss of cell viability in SU-DHL-6 and SU-DHL-10 cells treated with various concentrations of 1-L-MT in CCK-8 assay (Supplementary Fig. S2G, H). Taken together, these results suggested that MDM2-p53 was a crucial downstream factor of IDO1, and IDO1 might play tumor-promoting roles via the MDM2-p53 pathway, including the p53-related cell cycle and apoptotic pathway.

### IDO1 inhibition induced cell apoptosis and cell cycle arrest in DLBCL cells through activation of the p53 apoptotic pathway

To explore the possible mechanisms underlying 1-L-MT-induced apoptosis, we assessed the expression levels of the proapoptotic proteins BAX and PUMA and the antiapoptotic proteins BCL-2 and BCL-XL using WB. The proapoptotic protein BAX was increased in OCI-Ly3 cells after 5 mM 1-L-MT treatment, whereas no significant change was observed in the PUMA levels (Fig. 6G). In OCI-Ly10 cells, 1-L-MT increased the expression of PUMA compared with the control group, whereas the levels of BAX remained constant (Fig. 6H). RT-qPCR results demonstrated that the expression of BAX (Fig. 6M) and PUMA (Fig. 6N) was upregulated in OCI-Ly3 and OCI-Ly10 after treatment with 1-L-MT for 24 h. However, in *TP53* mutated cell lines of SU-DHL-6 and SU-DHL-10, 1-L-MT did not affect the protein expressions of BAX and PUMA (Supplementary Fig. S2I, J). Furthermore, WB results demonstrated that 1-L-MT decreased the levels of the antiapoptotic proteins BCL-2 and BCL-XL in both OCI-Ly3 and OCI-Ly10 cells (Fig. 6G, H). GSEA also confirmed that IDO1 inhibition resulted in apoptosis in OCI-Ly10 cells (NSE 1.8780538, *P* < 0.001, Fig. 6I).

WB was used to evaluate the expression levels of key cell cycle regulators, including p53 and p21. As shown in Fig. 6J, K, the expression of p53 was remarkably increased in OCI-Ly3 and OCI-Ly10 cells treated with 1-L-MT at 5 mM for 24 h. p21, a p53 transcriptional target, was prominently upregulated in OCI-Ly3 and OCI-Ly10 cells treated with 1-L-MT (Fig. 6J, K). Besides, we noted that p21 expression was not influenced by 1-L-MT in SU-DHL-6 and SU-DHL-10 cells (Supplementary Fig. S2I, J). Immunofluorescence staining also confirmed the upregulated p53 protein level in OCI-Ly3 cells treated with 1-L-MT at 5 mM for 24 h (Fig. 6L). RT-qPCR results demonstrated that the expression of p21 (Fig. 6O) was upregulated in OCI-Ly3 and OCI-Ly10 after treatment with 1-L-MT for 24 h. These results indicated that increased p53 and p21 protein expression in DLBCL cells treated with 1-L-MT may induce G2-M phase arrest.

**Fig. 7:**
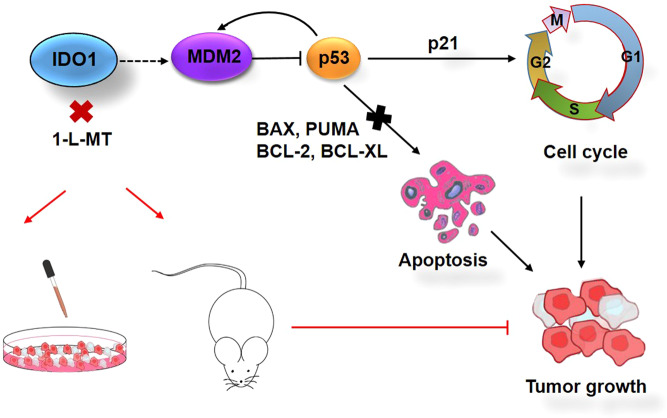
A model depicts the role of the IDO1-MDM2-p53 pathway in DLBCL growth. MDM2 and p53 act downstream of IDO1 to regulate tumor cell growth. This illustrates that IDO1 inhibition could activate the p53 pathway and induce cell cycle arrest and apoptosis by downregulating MDM2 expression in DLBCL cells.

As envisioned, the present results suggest that MDM2 and p53 are genes downstream of IDO1. 1-L-MT can activate the p53 pathway by suppressing MDM2 expression and inhibiting DLBCL cell growth by inducing cell cycle arrest and apoptosis (Fig. 7).

## Discussion

In the current study, we reported a crucial role of IDO1 in DLBCL tumor growth and that IDO1 overexpression was significantly correlated with poor outcomes in DLBCL patients. In addition, the IDO1 inhibitor reduced cell proliferation and induced cell cycle arrest and apoptosis in DLBCL cells. Besides, we demonstrated that the MDM2-p53 signaling pathway was downstream of IDO1 in DLBCL based on RNA-seq analysis. Furthermore, our results suggested that 1-L-MT activates the p53 pathway by suppressing MDM2 expression, inducing cell cycle arrest and apoptosis in DLBCL cells (Fig. 7).

Previous studies revealed that IDO1 was highly expressed in multiple tumor types, including lung cancer, breast cancer, colorectal cancer, and renal cell carcinoma [35–39], and many clinical trials are being carried out to test the strategy of the combination of IDO1 inhibitors with conventional treatments, including PD-1 antibodies, chemotherapy, and radiotherapy. Consistent with previous studies, our results revealed an increase in the expression level of IDO1 in DLBCL tissues compared with normal tissues. In addition, we confirmed the IDO1 expression levels in DLBCL tissues using GEO and TCGA datasets [18–20]. Our results found that the high expression of IDO1 was observed in tumor tissues and was associated with unfavorable outcomes, indicating that IDO1 may be a potential therapeutic biomarker. However, the correlation between IDO1 overexpression and clinical outcomes in DLBCL is unknown [6, 8]. We evaluated the association between IDO1 expression level and clinical outcome in 97 DLBCL patients who were enrolled in our hospital from 2006 to 2013. In this study, IDO1 overexpression was found to be significantly correlated with poor clinical features and outcomes. The above results were in good agreement with a previous study [6]. Nevertheless, the clinical efficacy of IDO1 inhibitors as single agents remains unclear, and the specific mechanisms by which IDO1 affects the role of immune cells and restrains the immune system remain unknown.

In pathway analysis, we used 1-L-MT, an IDO1 inhibitor [40, 41], to assess the antitumor effect of IDO1 inhibition in OCI-Ly3 and OCI-Ly10 cells and NOD/SCID mice. The safety of 1-L-MT in vitro and in vivo has been widely confirmed by various studies [42, 43]. We confirmed that 1-L-MT could inhibit DLBCL cell proliferation in vitro and suppress tumor growth in vivo. Our RNA-seq analysis revealed that MDM2 was downregulated while TP53 was upregulated in 1-L-MT-treated OCI-Ly10 cells compared with the control group. As expected, RT-qPCR validation confirmed the RNA-seq results. GSEA and KEGG pathway analyses showed DEGs involved in the p53 signaling pathway in 1-L-MT-treated OCI-Ly10 cells. These results indicated that IDO1 is an upstream gene of the MDM2-p53 signaling pathway.

The tumor suppressor p53 induces cell cycle arrest, apoptosis, senescence, and autophagy under diverse cellular stresses, such as DNA damage [44, 45]. TP53 mutations have been reported in 22–24% of DLBCL patients [46, 47], and TP53 has frequently been found to be wild-type in DLBCL. A previous study showed that MDM2 can negatively regulate p53 and promote its polyubiquitination [48]. Furthermore, the dysregulation of MDM2-p53 is one of the critical mechanisms to promote DLBCL. In the present study, the result of WB confirmed the IDO1-MDM2-p53 regulatory relationship. IDO1 inhibition decreased MDM2 expression and increased levels of p53 in OCI-Ly3 and OCI-Ly10 cells. Moreover, MDM2 inhibitor-treated DLBCL cells showed that the expression of MDM2 and p53 was upregulated but did not affect the expression of IDO1.

Cell cycle and apoptosis progression are principal steps for tumor growth. As the first transcriptional target of p53, the cyclin-dependent kinase (CDK) inhibitor p21 can lead to cell cycle arrest by binding to cyclin/CDK complexes [49, 50]. Our results showed that IDO1 inhibition induced G2/M phase arrest through upregulation of p21 and p53 expression in DLBCL cells, which is in good agreement with previous studies [50–52]. These findings reveal that cycle arrest may be one of the possible molecular mechanisms for the effects of IDO1 inhibition on DLBCL growth. As transcriptional targets for activation by p53, the proapoptotic proteins PUMA and BAX presumably participate in cell apoptosis regulated by the p53 apoptotic pathway [53, 54]. Following these findings, our results showed that BAX upregulation was observed in 1-L-MT-treated OCI-Ly3 cells, whereas 1-L-MT-treated OCI-Ly10 cells increased PUMA compared with that in control cells. Other studies also showed a similar role of PUMA and BAX in cell apoptosis [50, 55]. Furthermore, the antiapoptotic proteins BCL2 and BCL-XL inhibit p53-induced apoptosis [56]. In our study, IDO1 inhibitor-treated OCI-Ly3 and OCI-Ly10 cells showed decreased BCL-XL and BCL2 levels. Similar changes in MDM2 inhibitor-treated DLBCL cells have been observed [50]. These data suggested that IDO1 inhibition can suppress DLBCL growth by inducing cell cycle arrest and apoptosis through the activated p53 pathway.

In conclusion, our results suggested that IDO1 was upregulated in DLBCL and that IDO1 overexpression was associated with poor clinical outcomes. In this study, we found that the MDM2-p53 pathway was mediated by IDO1. IDO1 inhibition decreased MDM2 and increased p53 expression, which induced cell cycle arrest and cell apoptosis through transcriptional activation of p21 and other p53 target genes, such as PUMA and BAX. Our study revealed that IDO1 is essential for the proliferation of DLBCL cells and that it may be a potential therapeutic target and provide new insight to develop a promising strategy by combining IDO1 with conventional therapies for the treatment of DLBCL.

## Supplementary information

co-author agreement file

Supplementary Figure S1

Supplementary Figure S2

Supplementary Figure S3

Supplementary Figure S4

Supplementary Table S1

Supplementary Table S2

check list

## Data Availability

All data needed to evaluate the conclusions in the paper are present in the paper and/or the Supplementary materials. The authenticity of this article has been validated by uploading the key raw data to the Research Data Deposit (RDD) public platform (www.researchdata.org.cn), with the approval RDD number as RDDB2022159763.
