# Supplementary figures and images for: IDO1 plays a tumor-promoting role via MDM2-mediated suppression of the p53 pathway in diffuse large B-cell lymphoma
Source: Cell Death Dis. 2022 Jun 27;13(6):572. doi: 10.1038/s41419-022-05021-2 (PMC9237101; doi:10.1038/s41419-022-05021-2)

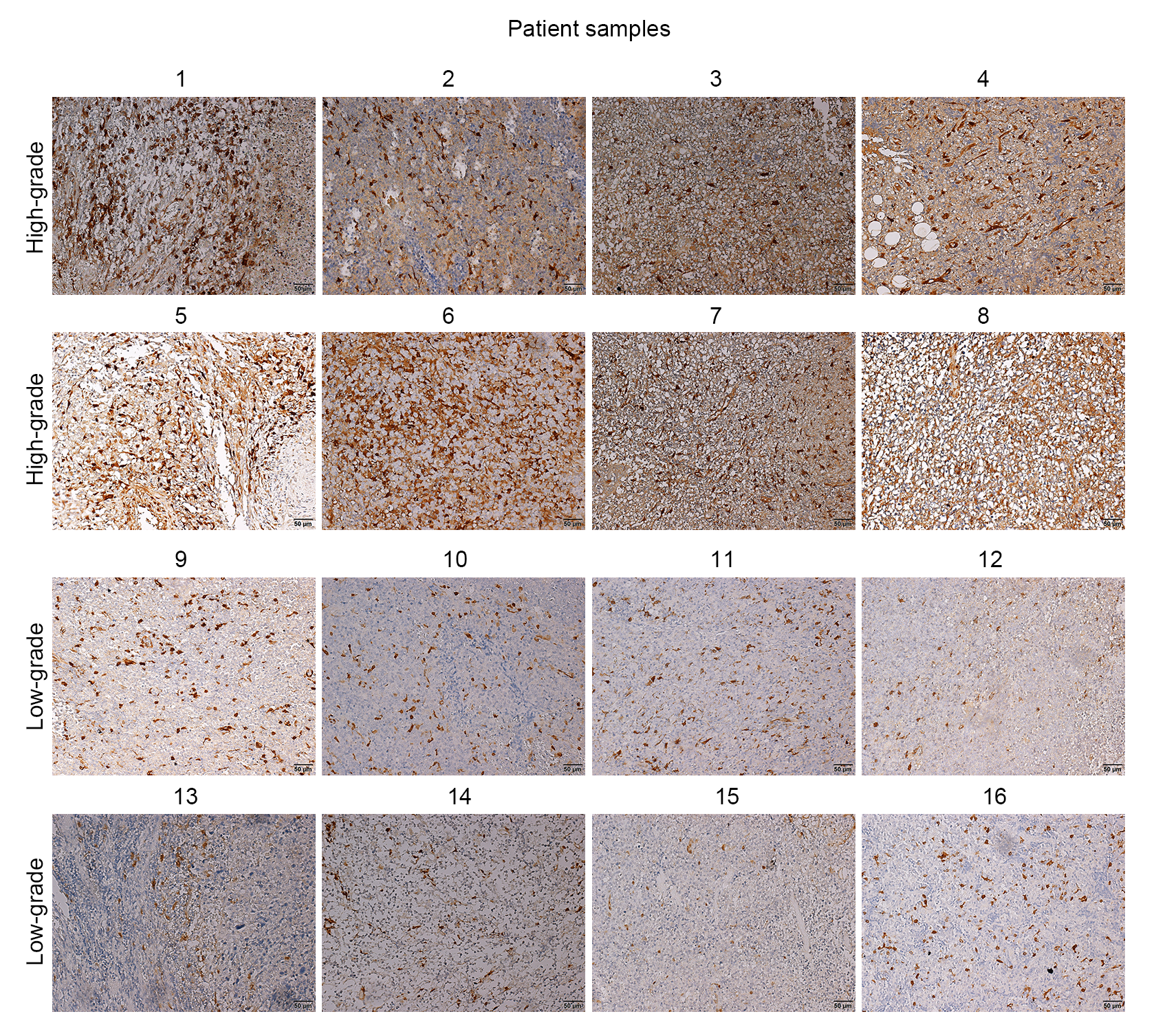

Supplement: Supplementary file 2 — Supplementary Figure S1 [file 41419_2022_5021_MOESM2_ESM.tif]

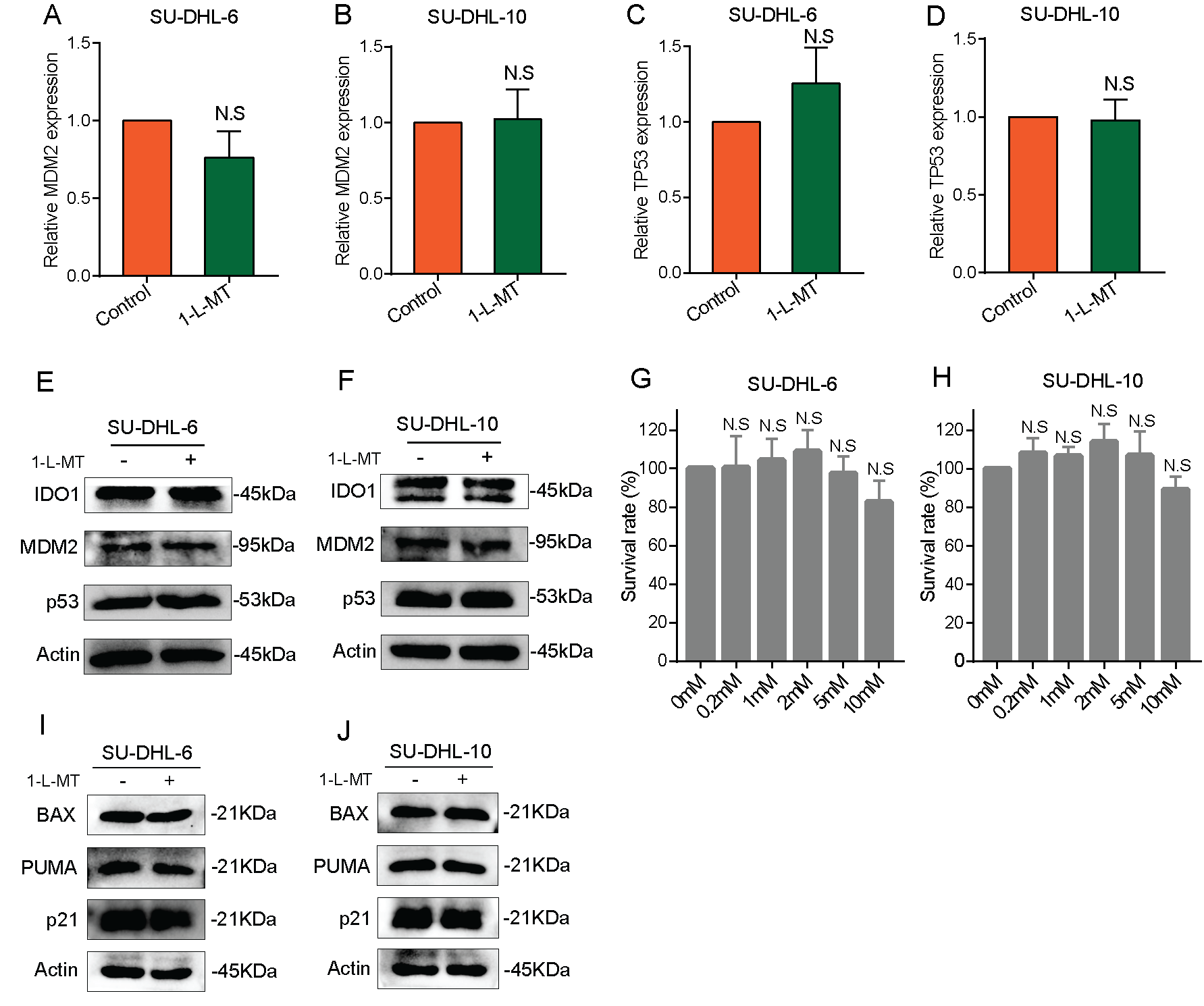

Supplement: Supplementary file 3 — Supplementary Figure S2 [file 41419_2022_5021_MOESM3_ESM.tif]

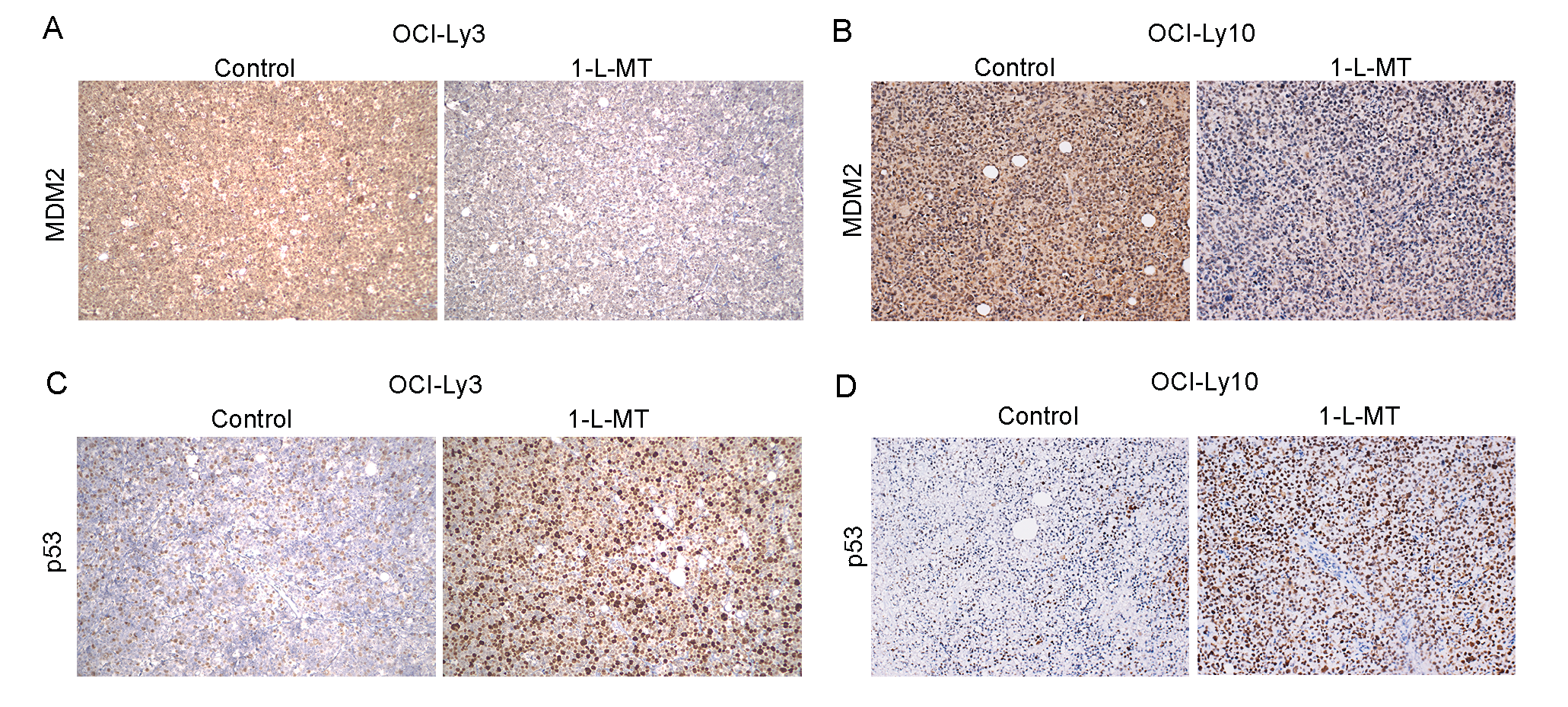

Supplement: Supplementary file 4 — Supplementary Figure S3 [file 41419_2022_5021_MOESM4_ESM.tif]
