# Supplementary material for: IDO1 plays a tumor-promoting role via MDM2-mediated suppression of the p53 pathway in diffuse large B-cell lymphoma
Source: Cell Death Dis. 2022 Jun 27;13(6):572. doi: 10.1038/s41419-022-05021-2 (PMC9237101; doi:10.1038/s41419-022-05021-2)

Figure 3C: WB results.
Repeat 1

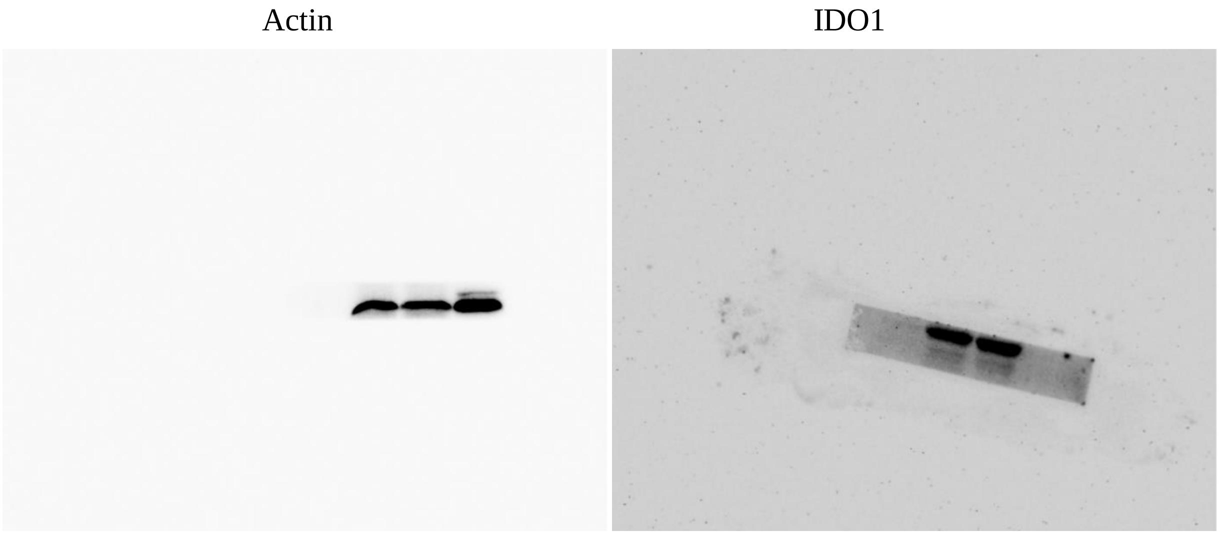

Figure 3C: WB results.
Repeat 2

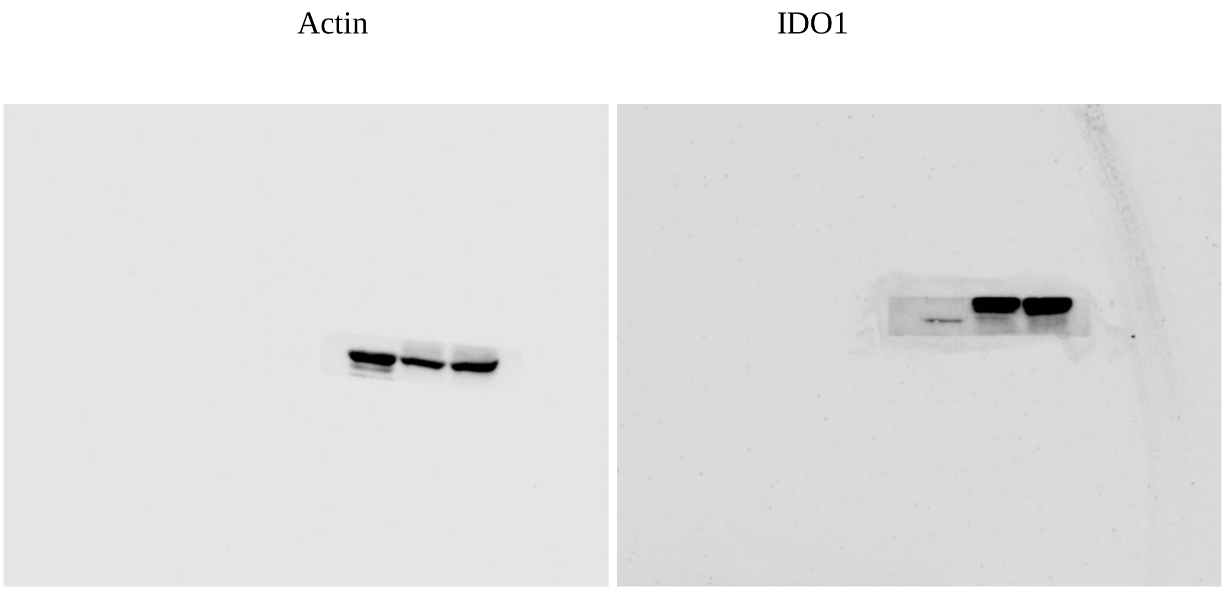

Figure 3C: WB results.
Repeat 3

Actin IDO1

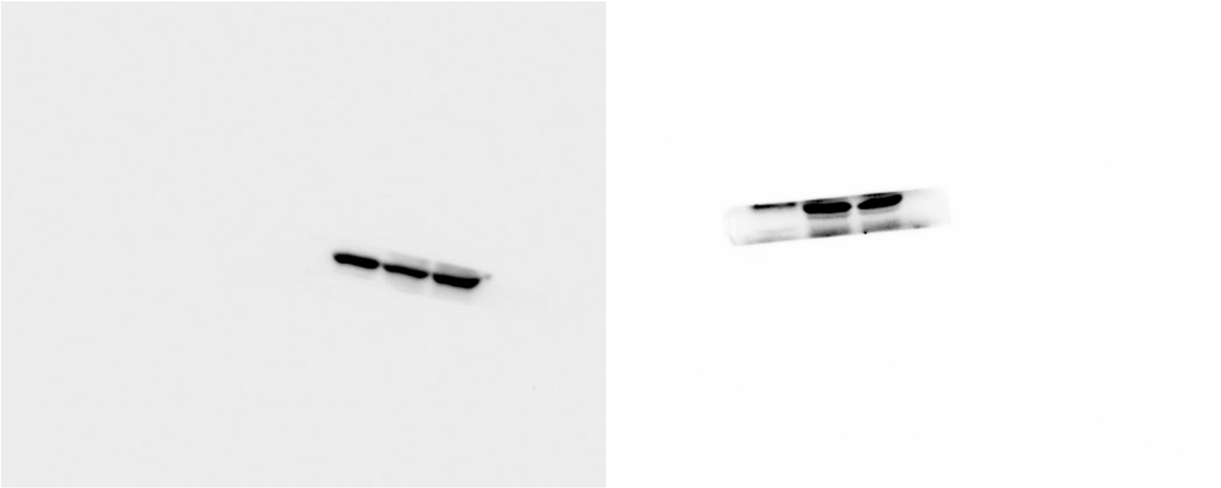

Figure 6C: WB results
Repeat 1

IDO1

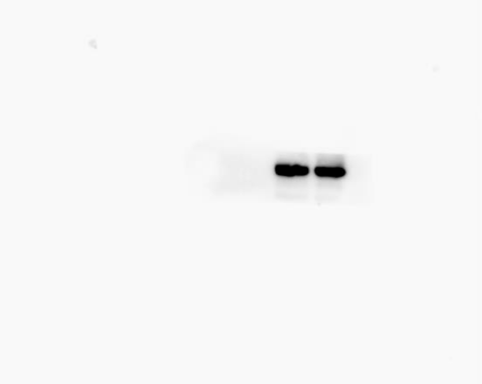

MDM2

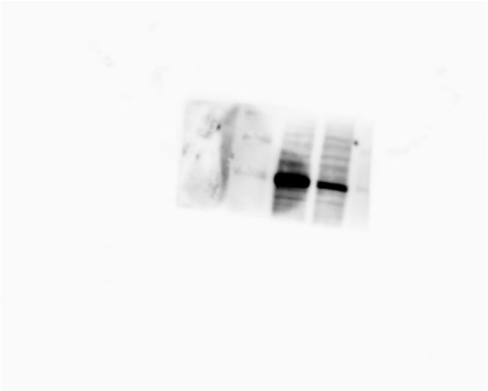

p53

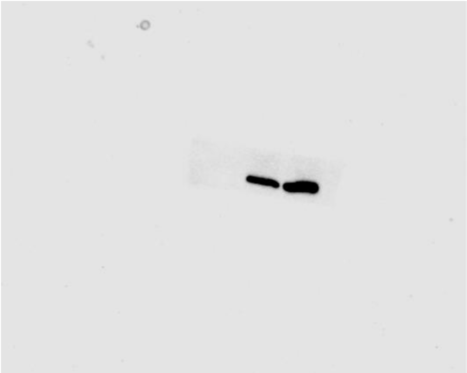

Actin

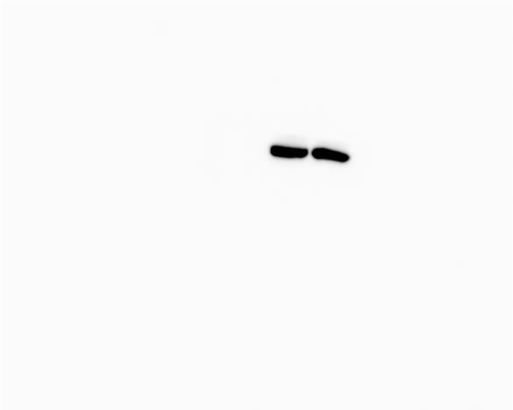

Figure 6C: WB results
Repeat 2

IDO1

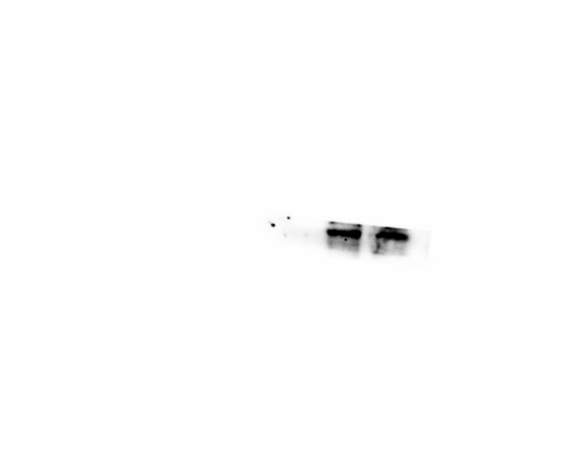

MDM2

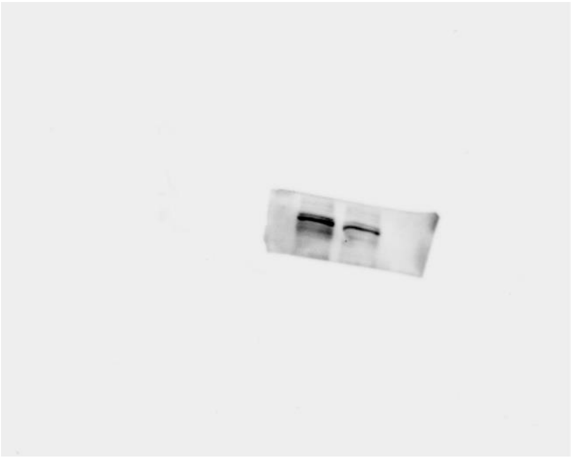

p53

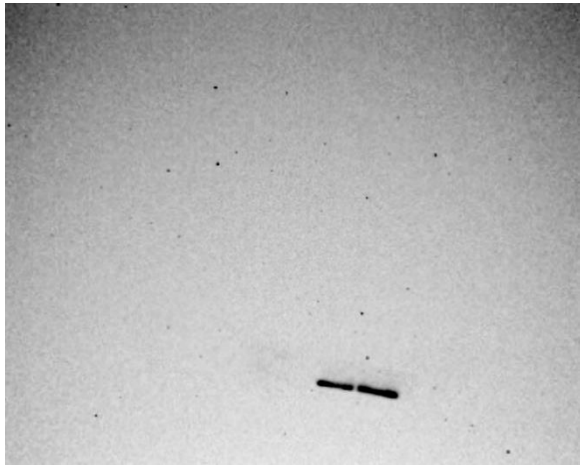

Actin

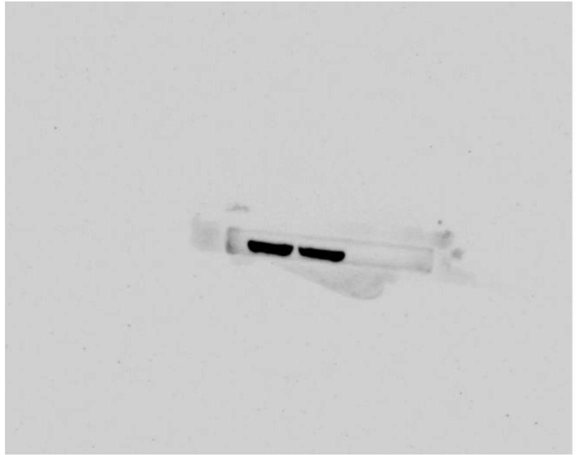

Figure 6C: WB results
Repeat 3

IDO1

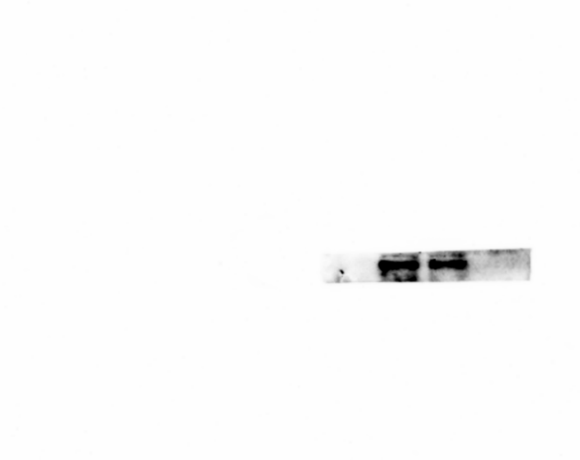

MDM2

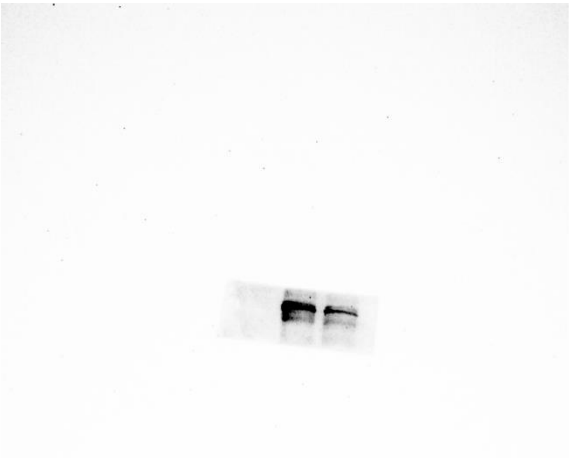

p53

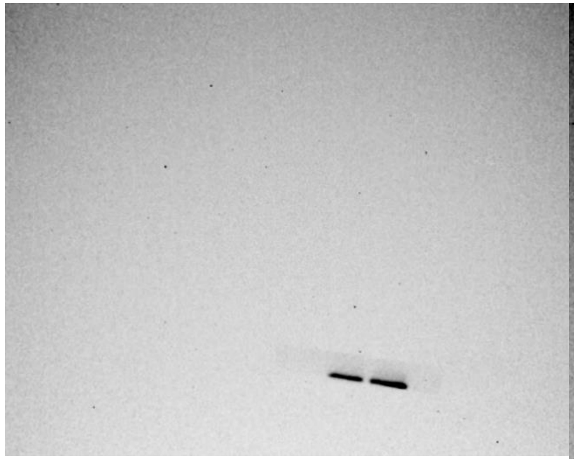

Actin

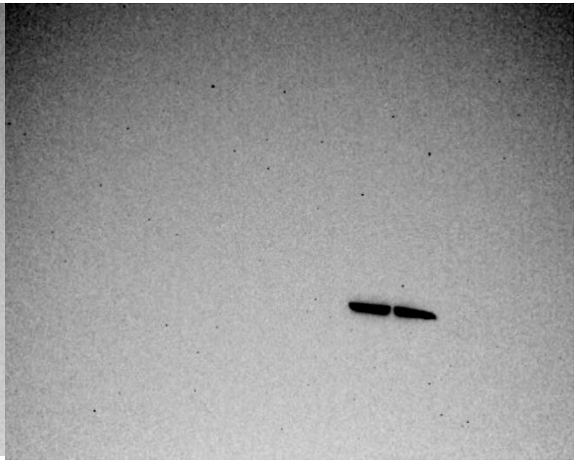

Figure 6D: WB results
Repeat 1

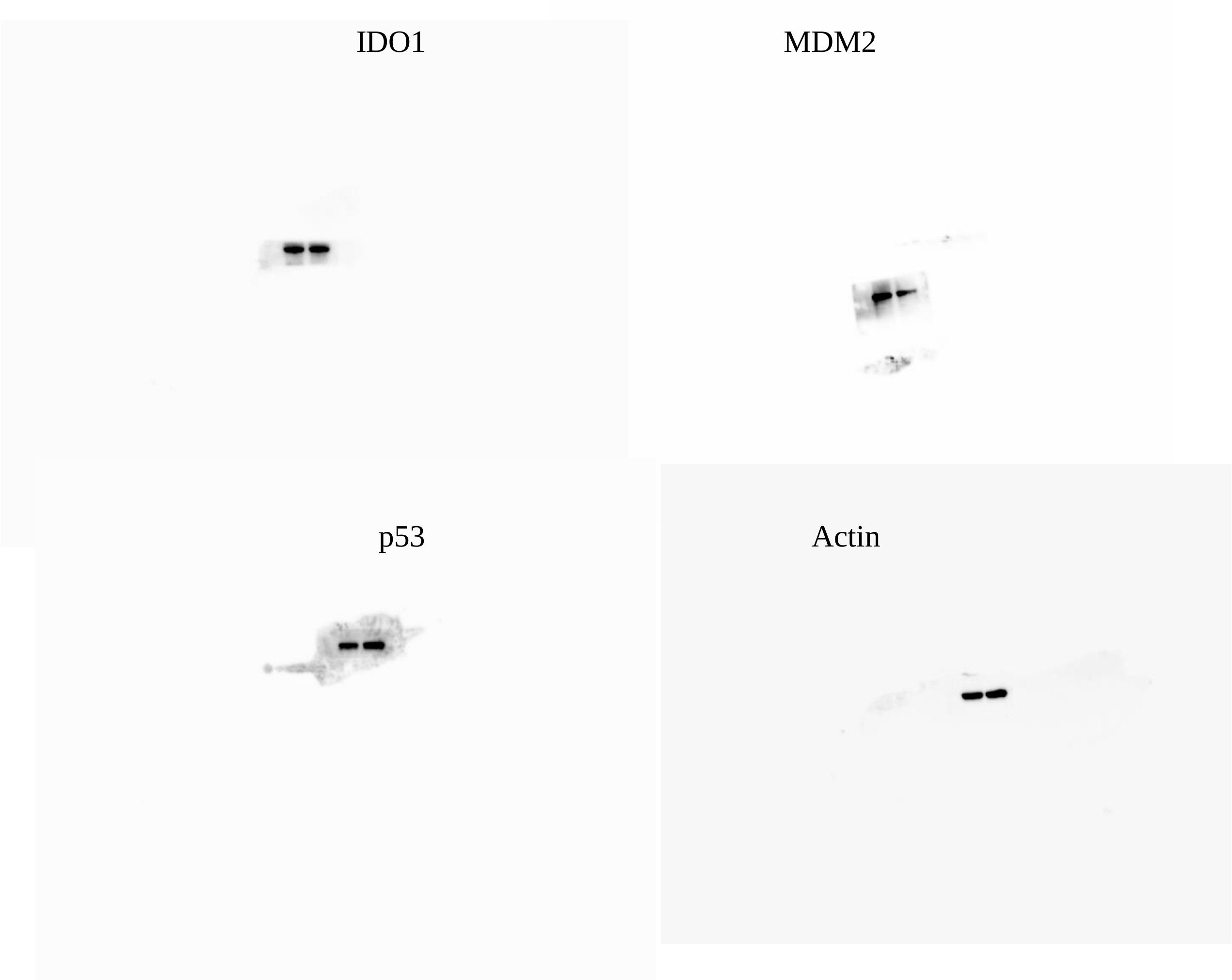

Figure 6D: WB results
Repeat 2

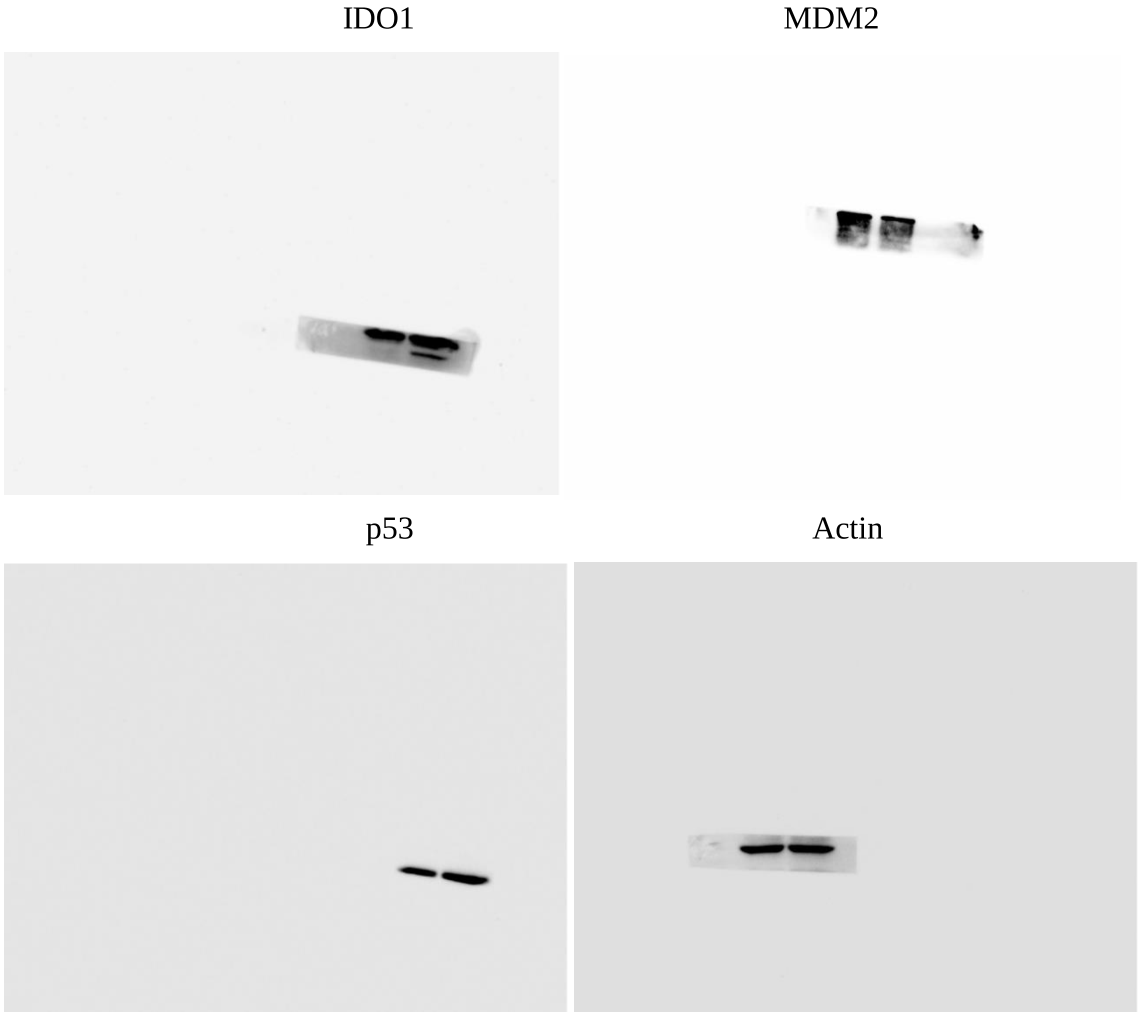

Figure 6D: WB results
Repeat 3

IDO1

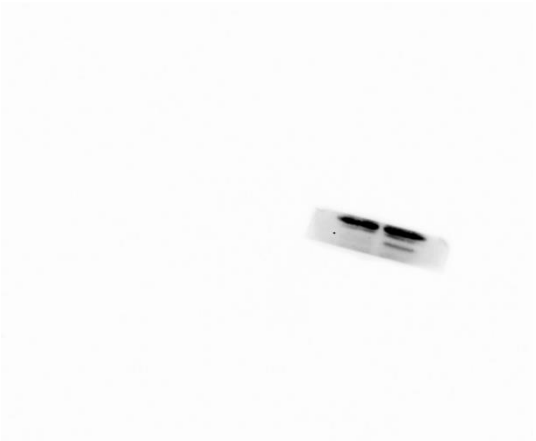

MDM2

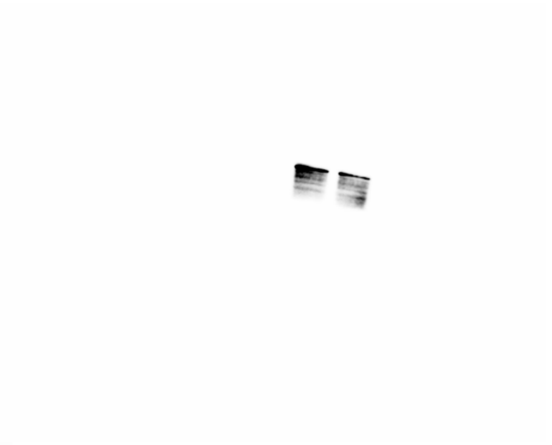

p53

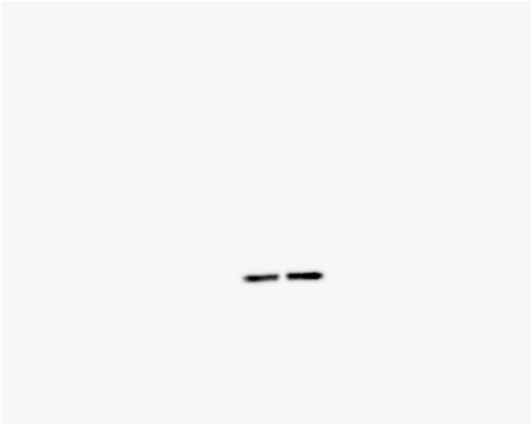

Actin

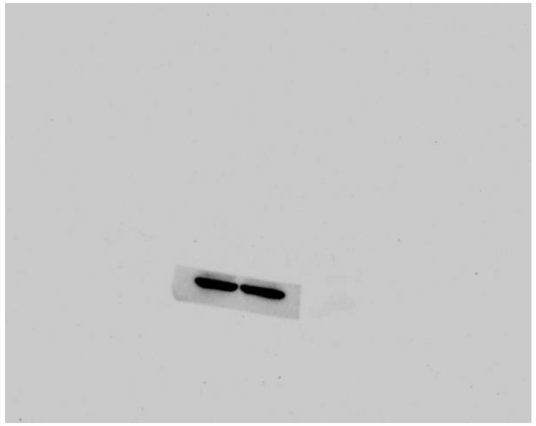

Figure 6E: WB results
Repeat 1

IDO1

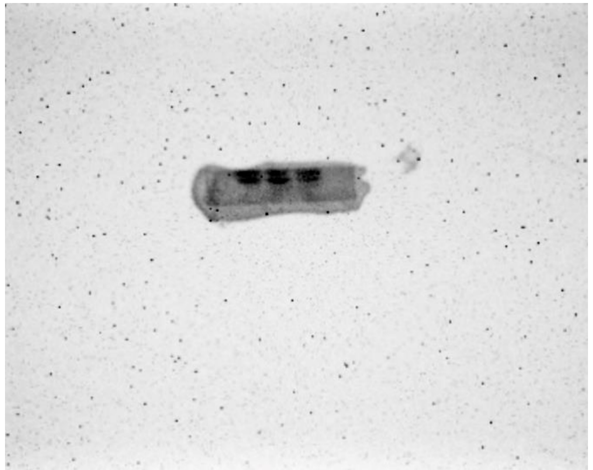

MDM2

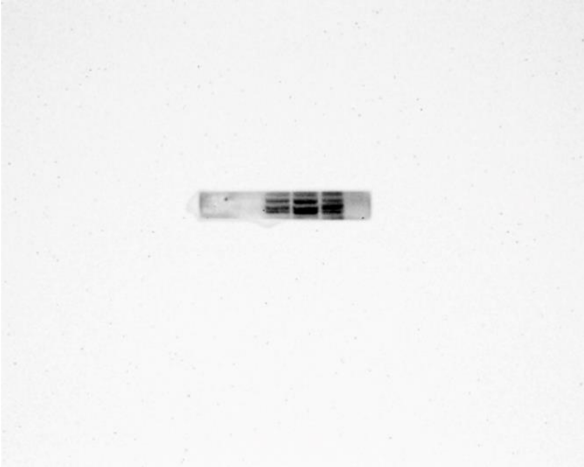

p53

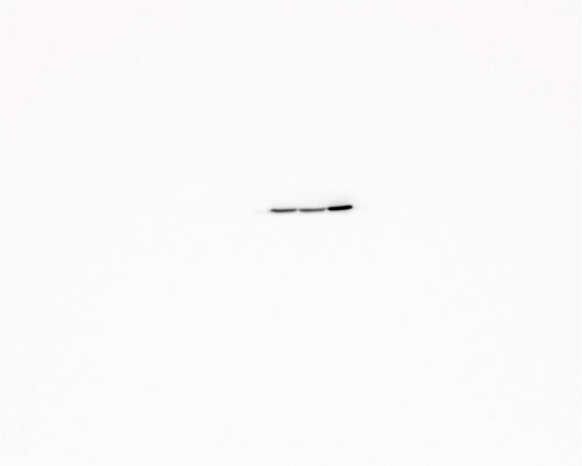

Actin

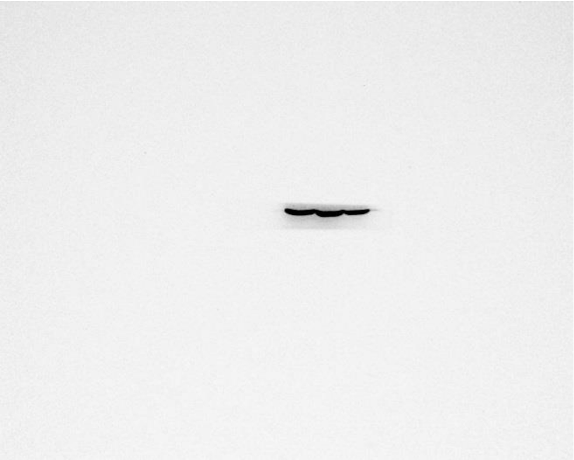

Figure 6E: WB results
Repeat 2

IDO1 MDM2

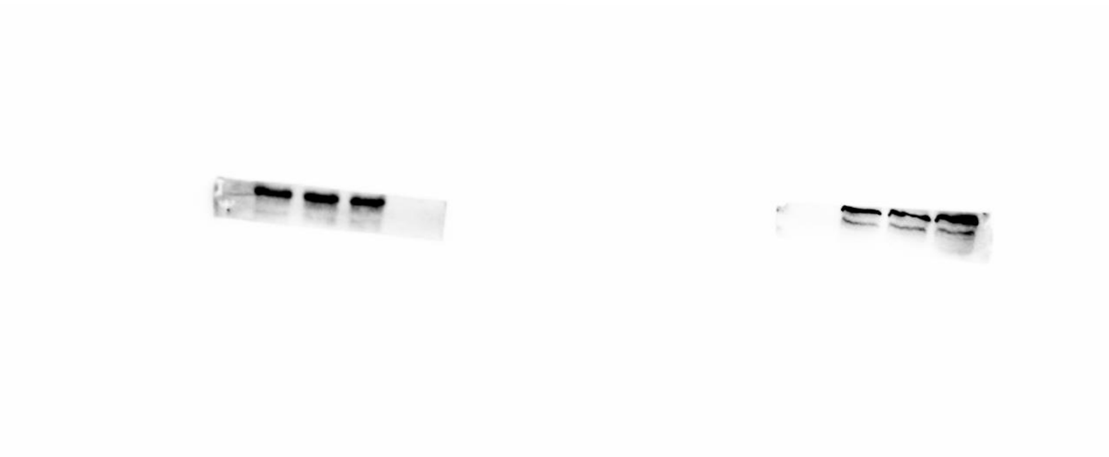

p53 Actin

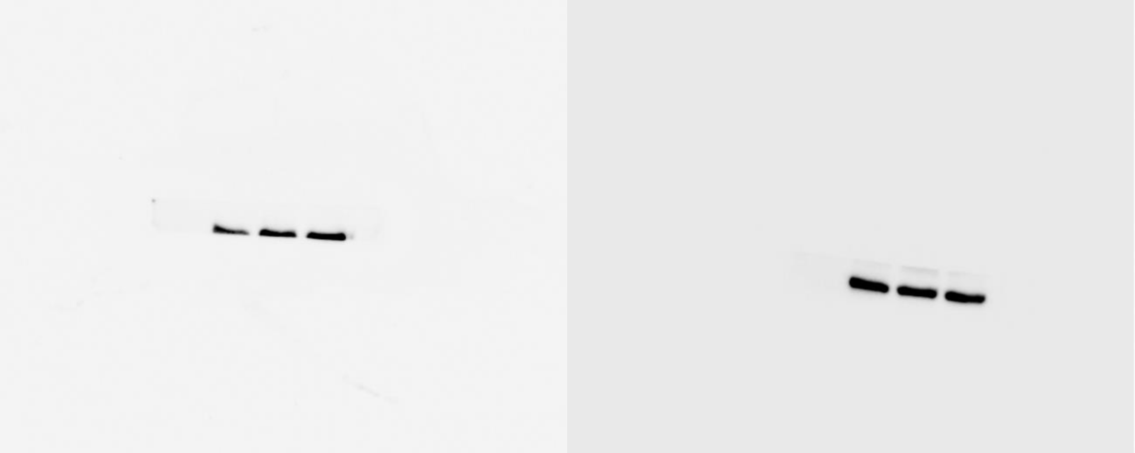

Figure 6E: WB results
Repeat 3

IDO1

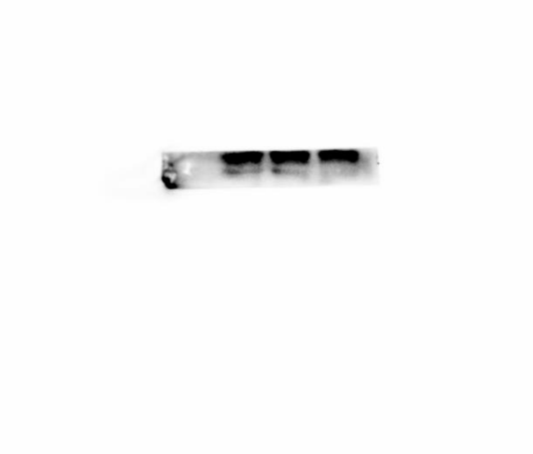

MDM2

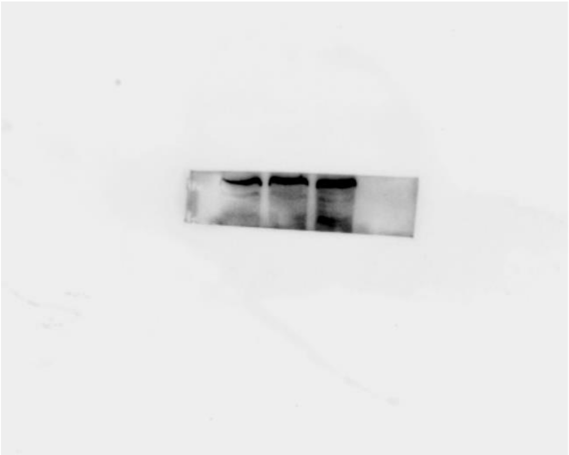

p53

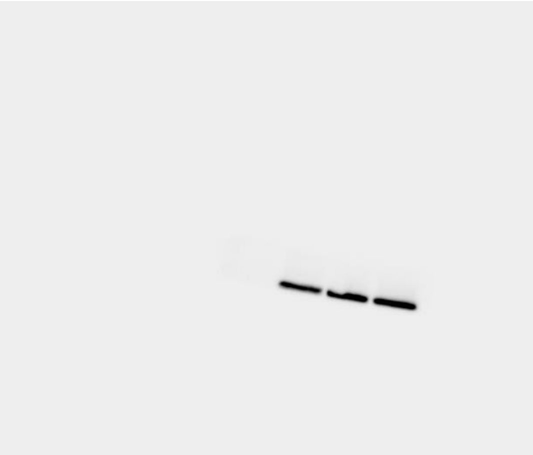

Actin

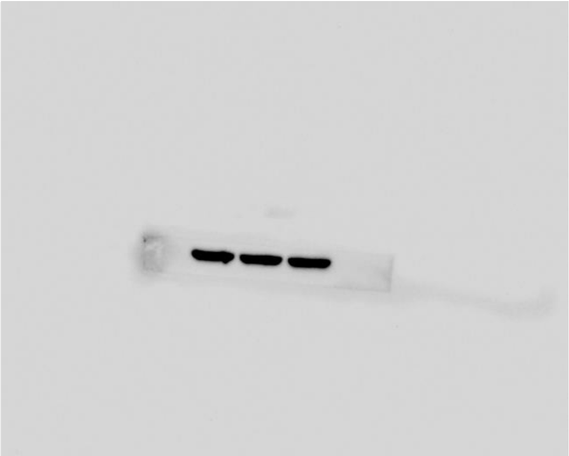

Figure 6F: WB results

Repeat 1

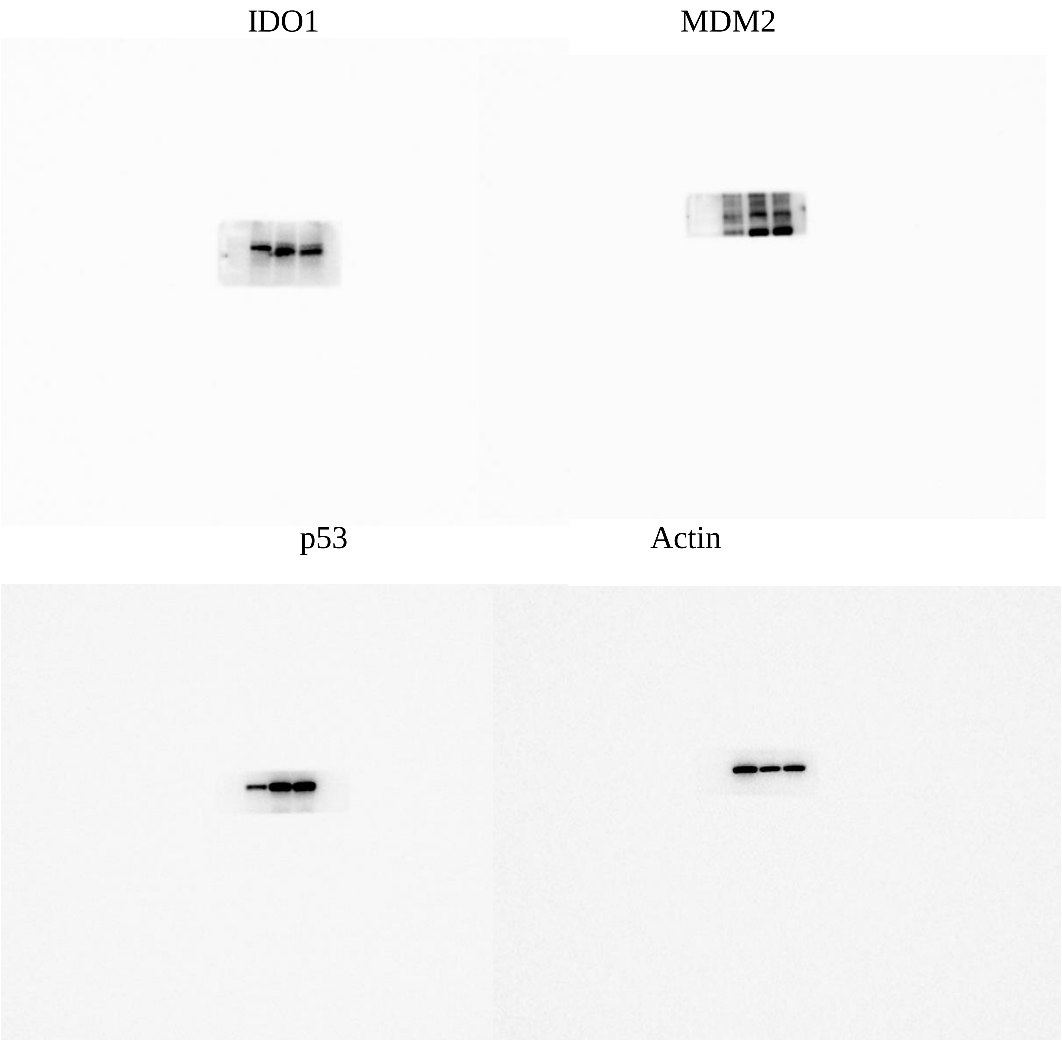

Figure 6F: WB results
Repeat 2

IDO1

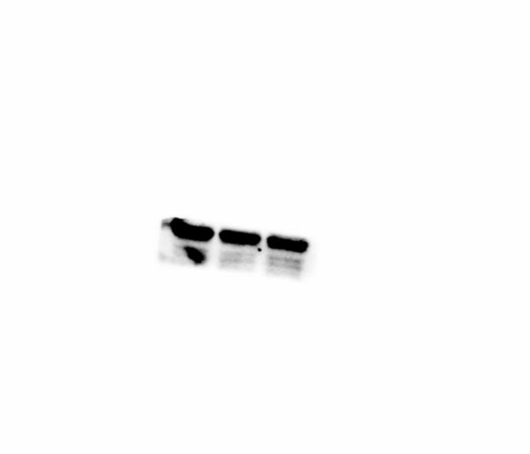

MDM2

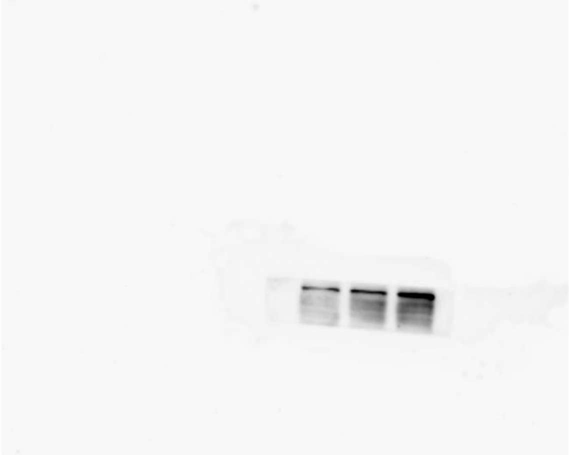

p53

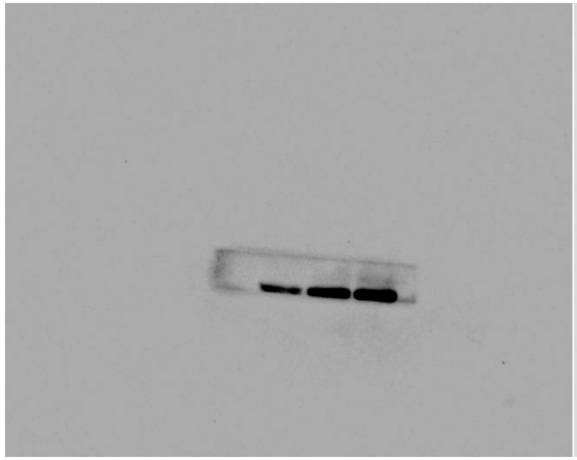

Actin

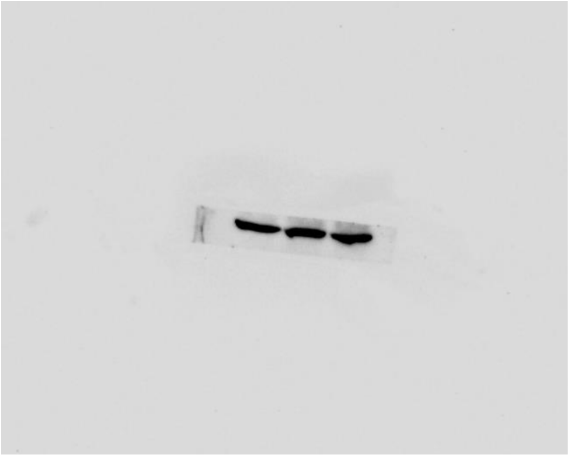

Figure 6F: WB results
Repeat 3

IDO1 MDM2

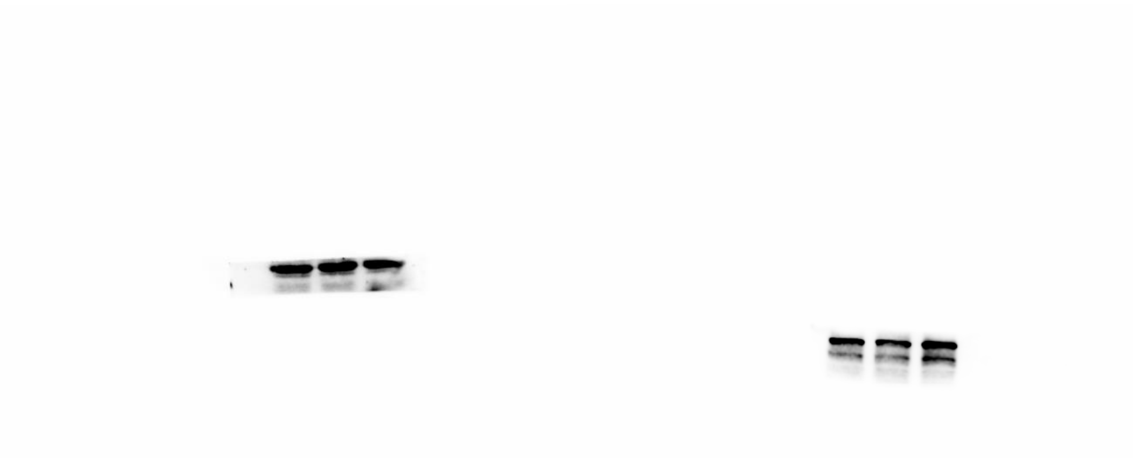

p53 Actin

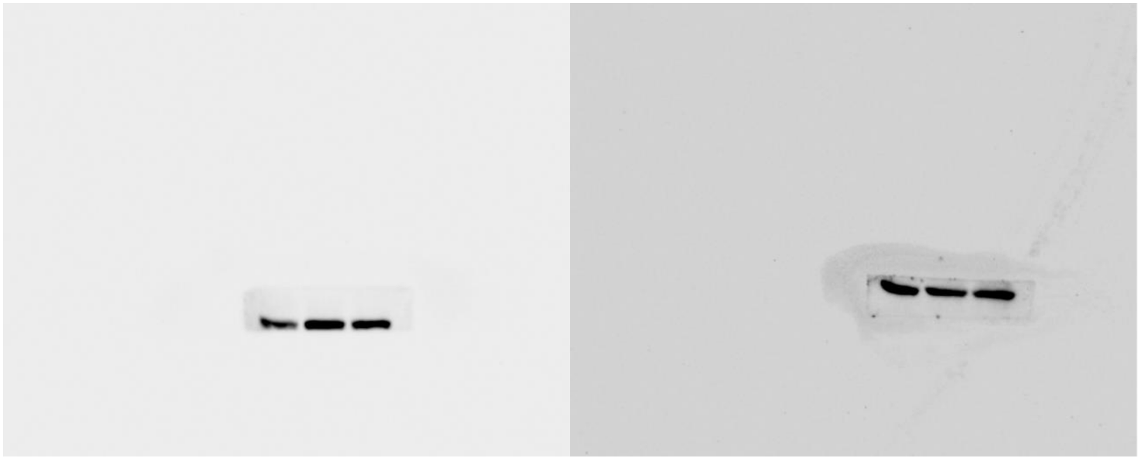

Figure 6G: WB results
Repeat 1

BAX

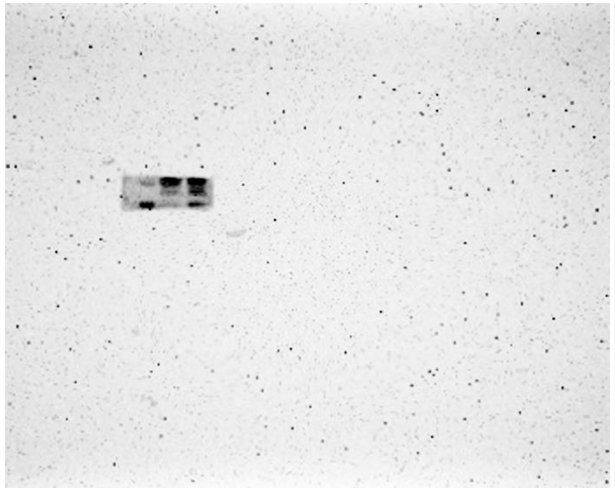

PUMA

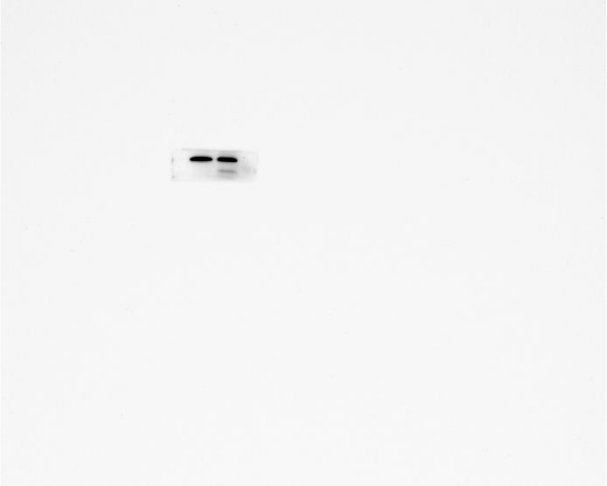

BCL-XL

BCL2

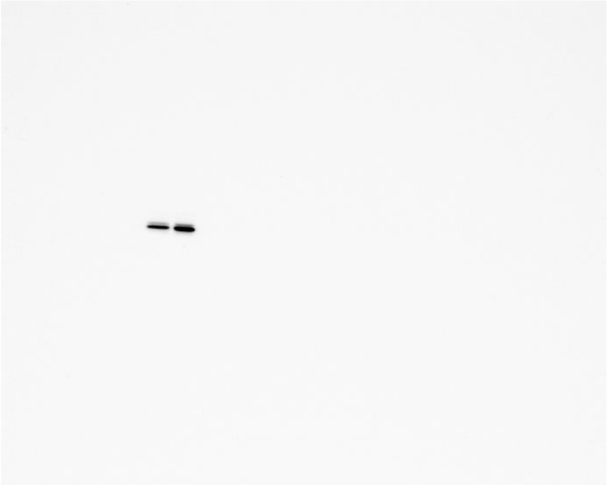

Actin

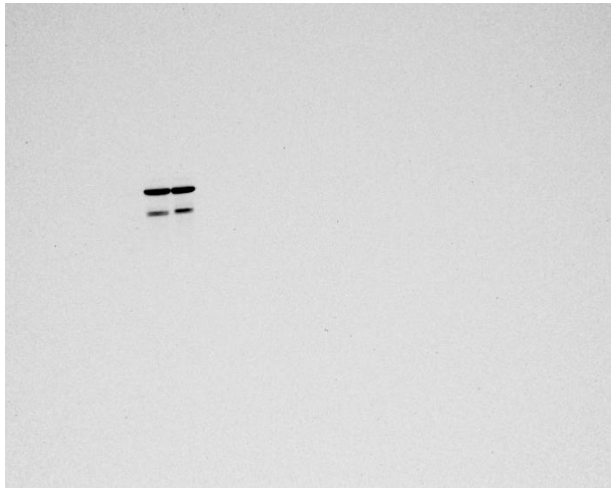

Figure 6G: WB results
Repeat 2

BAX

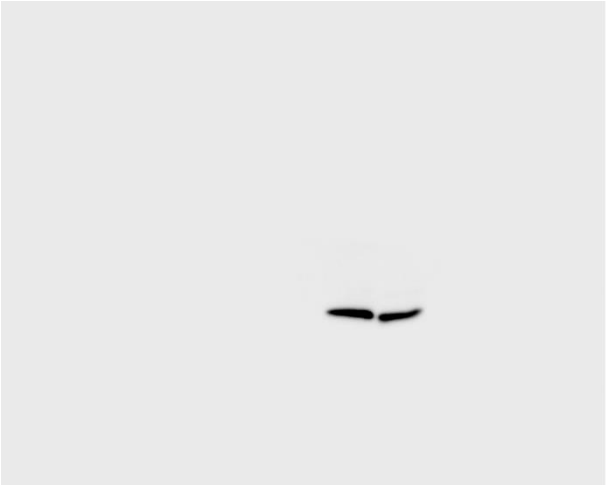

PUMA

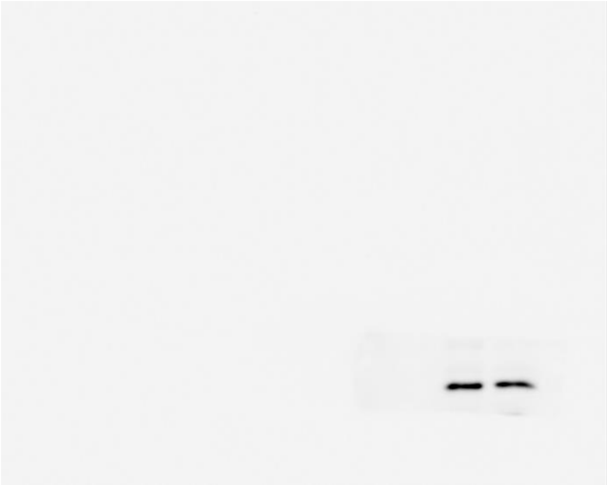

BCL-XL

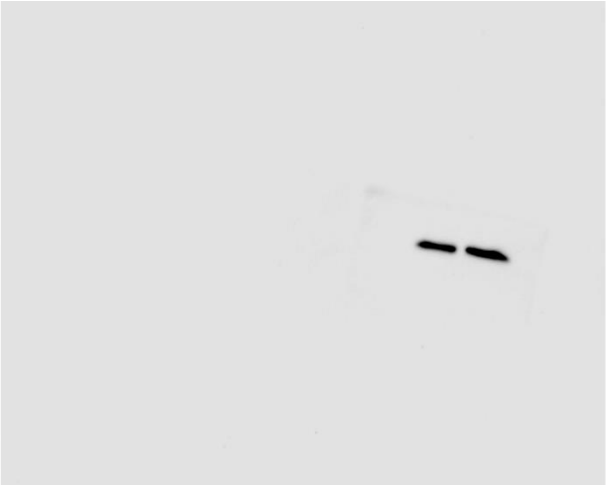

BCL2

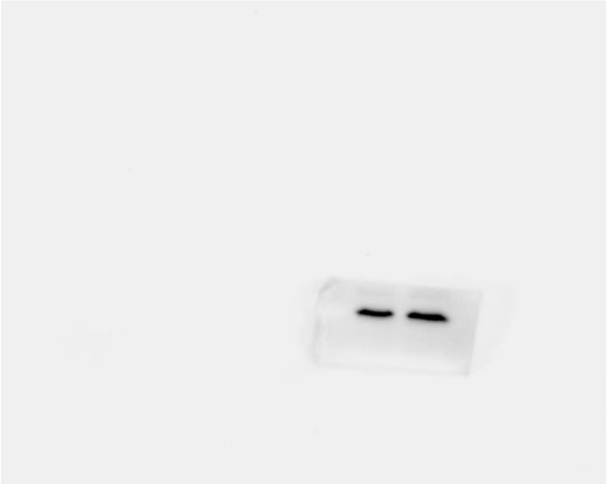

Actin

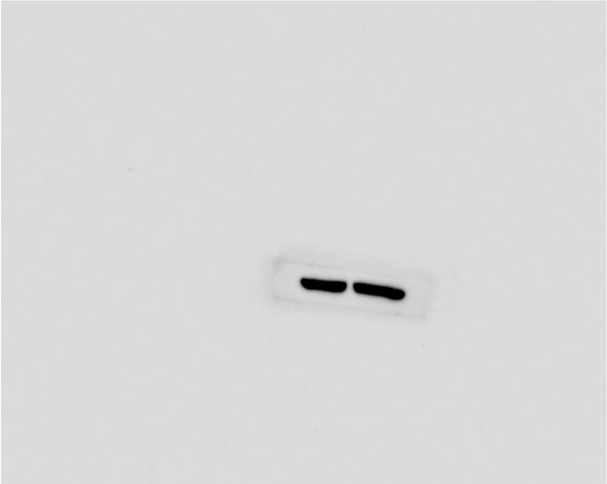

Figure 6G: WB results
Repeat 3

BAX

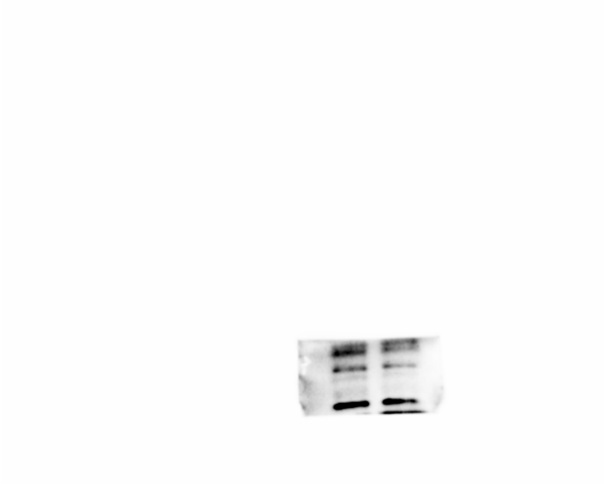

PUMA

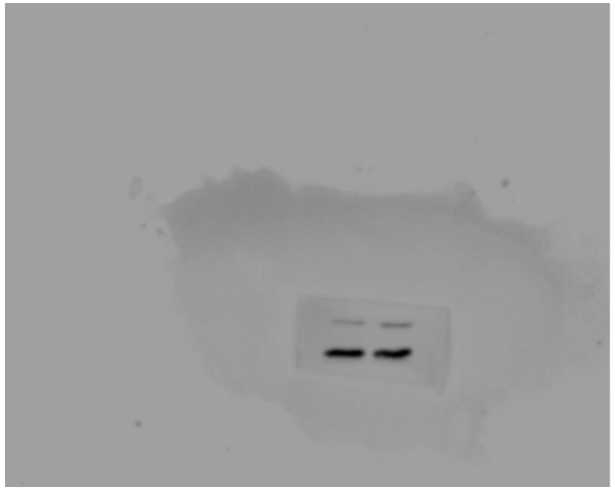

BCL-XL

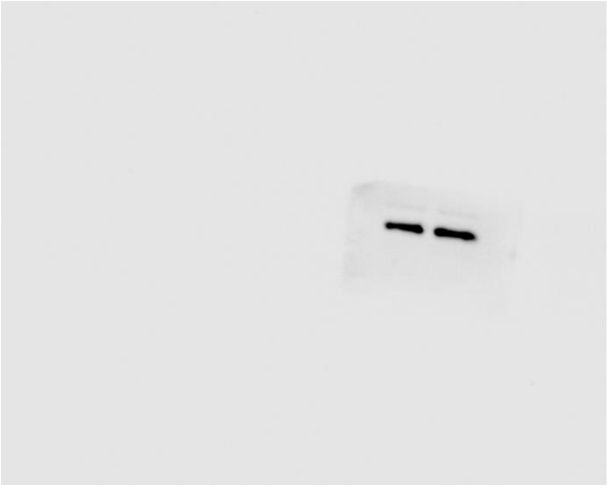

BCL2

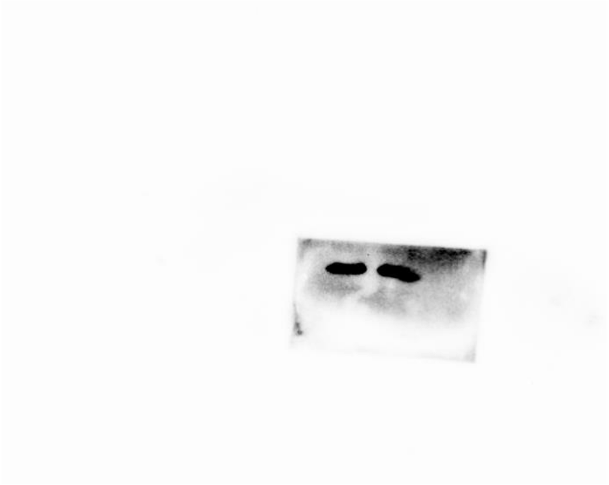

Actin

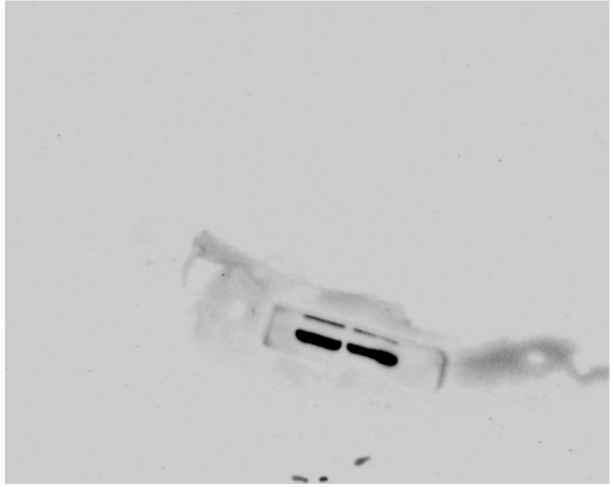

Figure 6H: WB results
Repeat 1

BAX

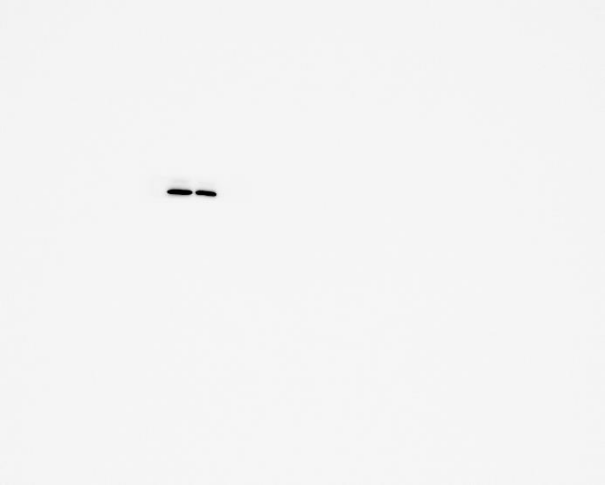

PUMA

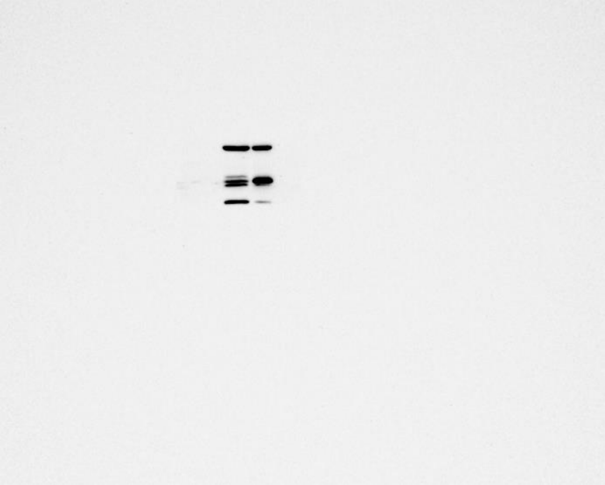

BCL-XL

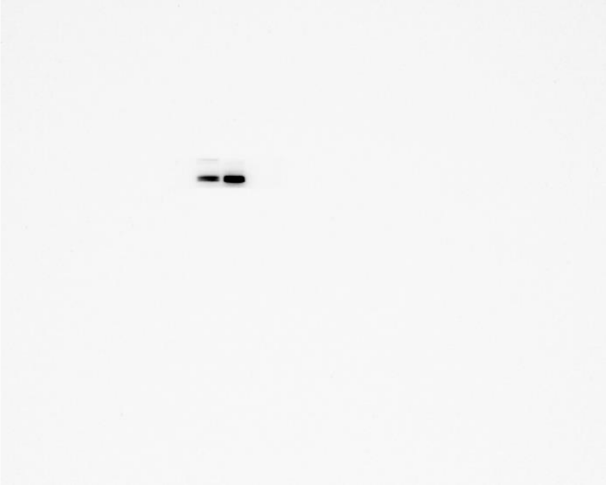

BCL2

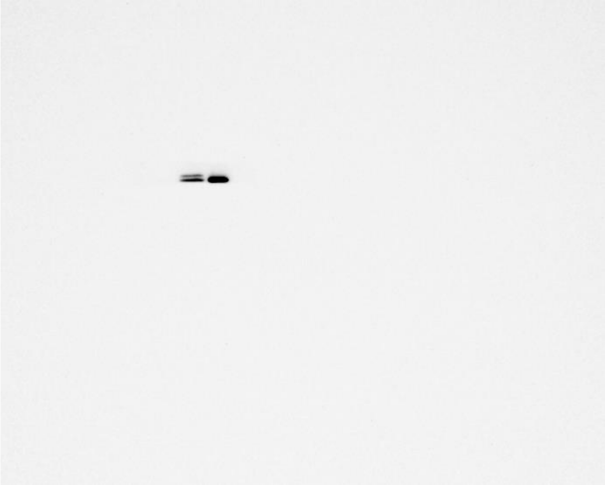

Actin

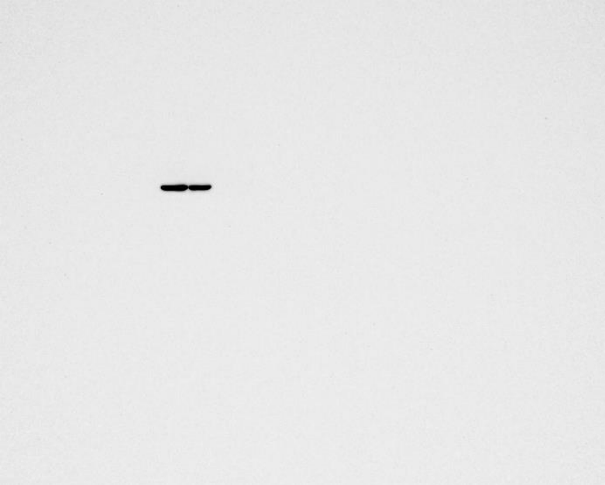

Figure 6H: WB results
Repeat 2

BAX

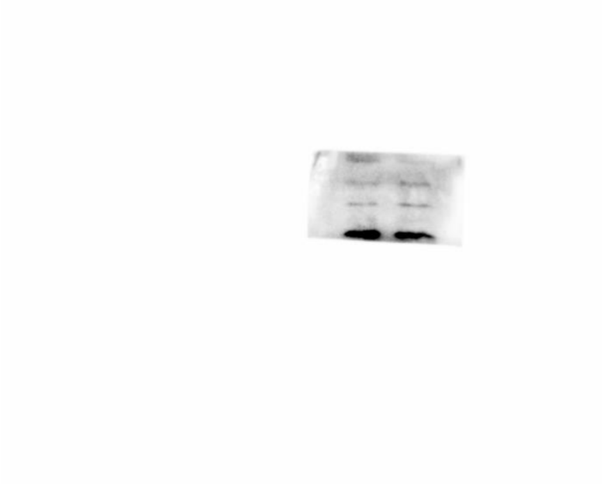

PUMA

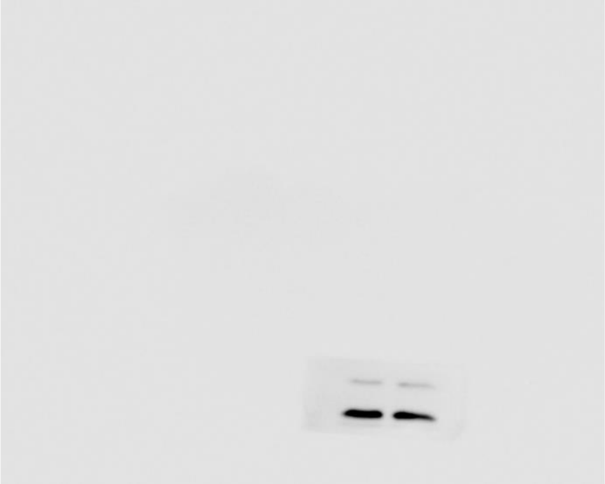

BCL-XL

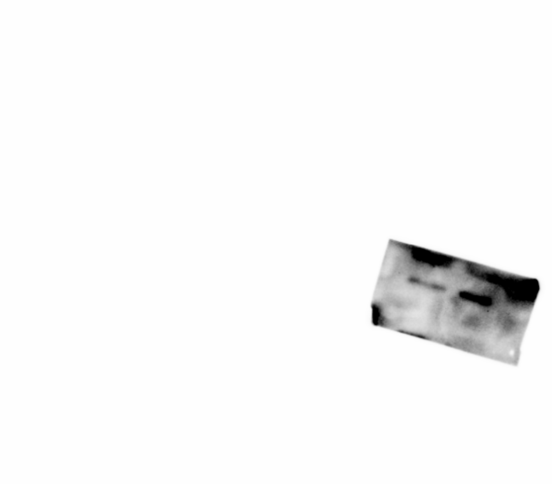

BCL2

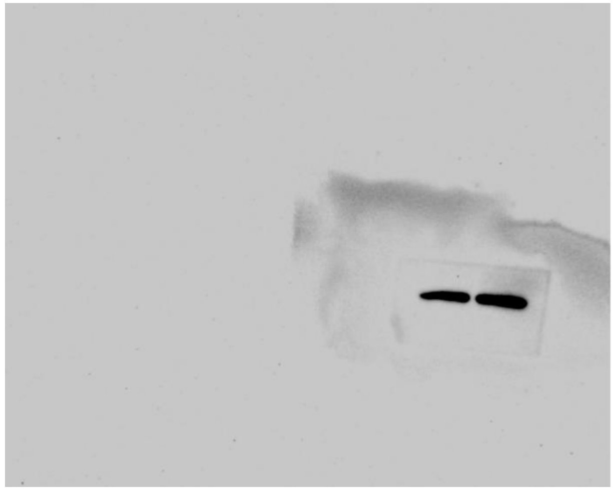

Actin

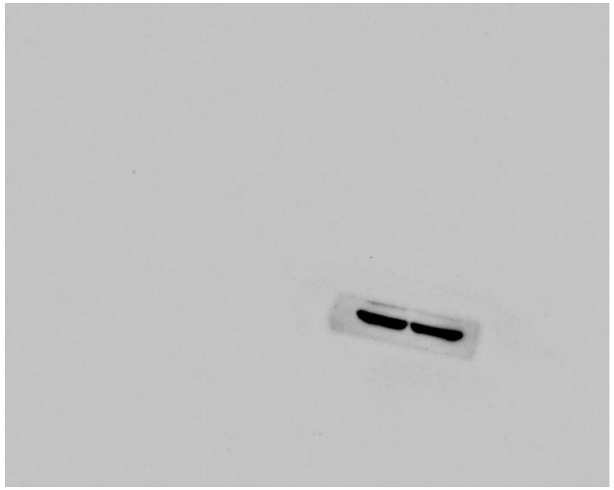

Figure 6H: WB results
Repeat 3

BAX

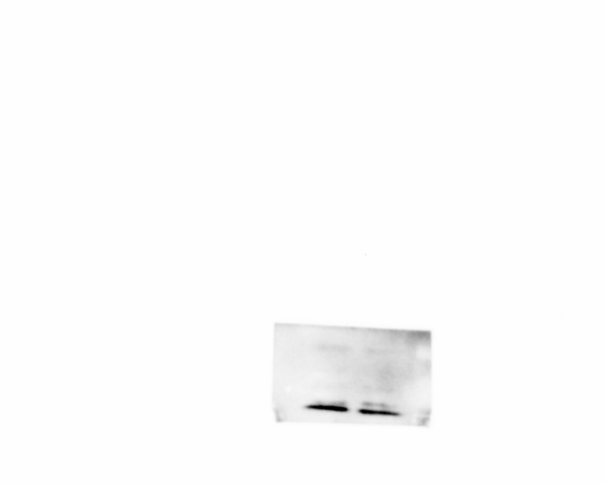

PUMA

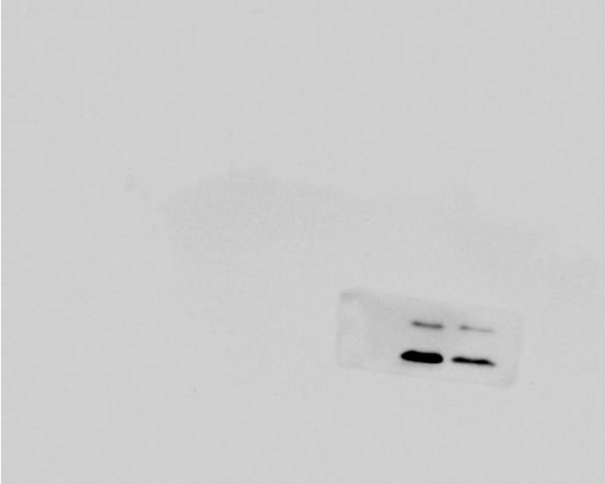

BCL-XL

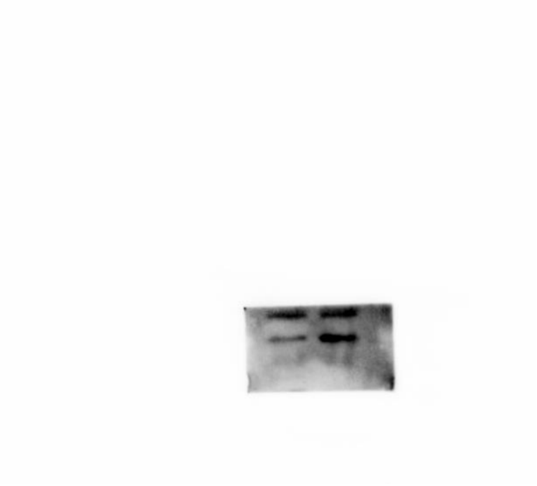

BCL2

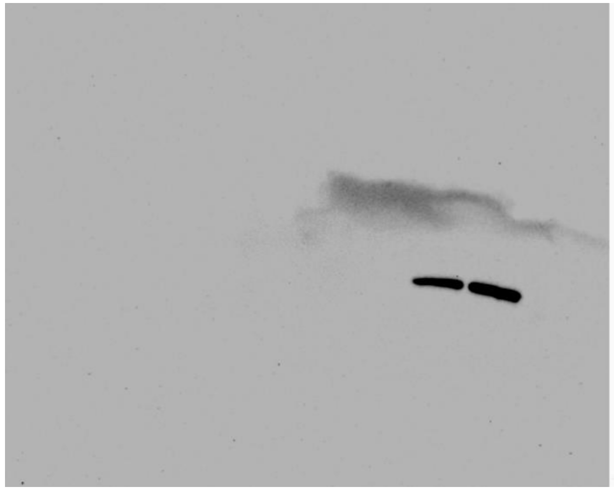

Actin

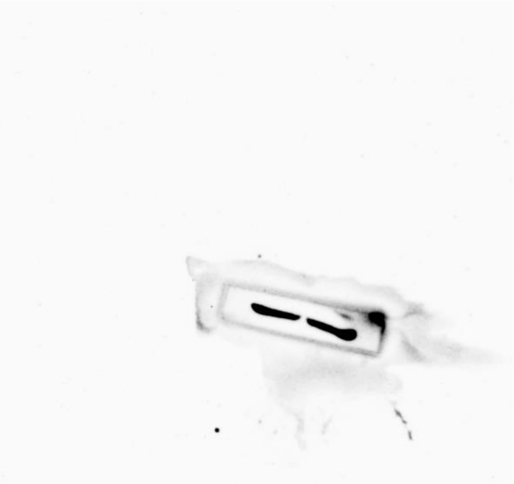

Figure 6J: WB results
Repeat 1

p53

p21

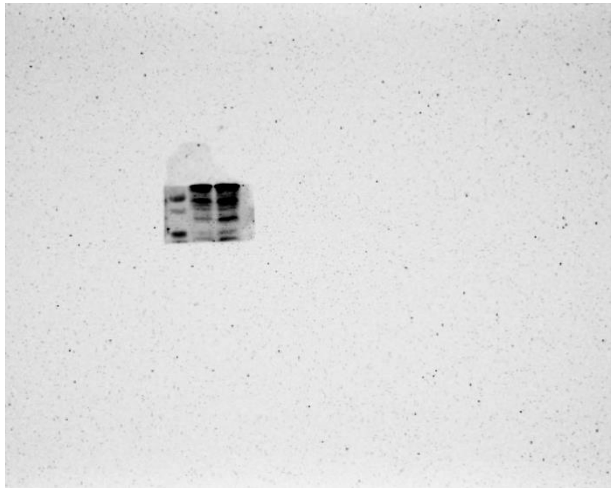

Actin

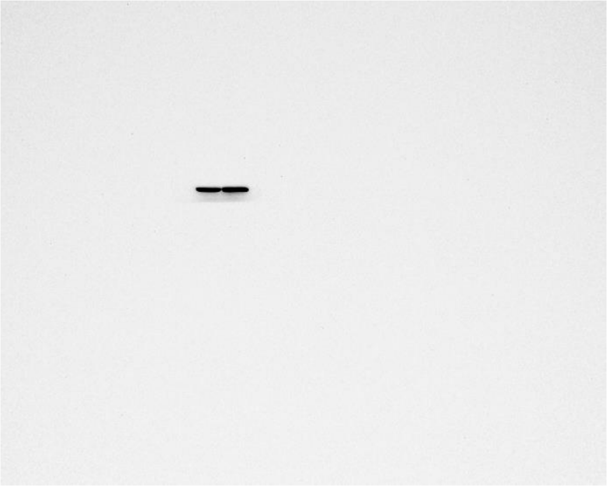

Figure 6J: WB results
Repeat 2

p53

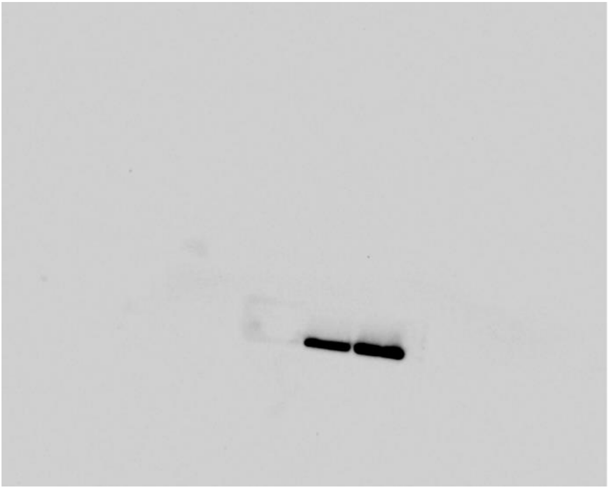

p21

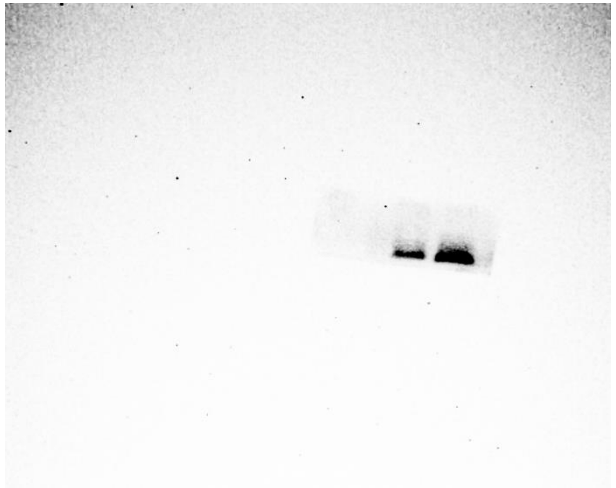

Actin

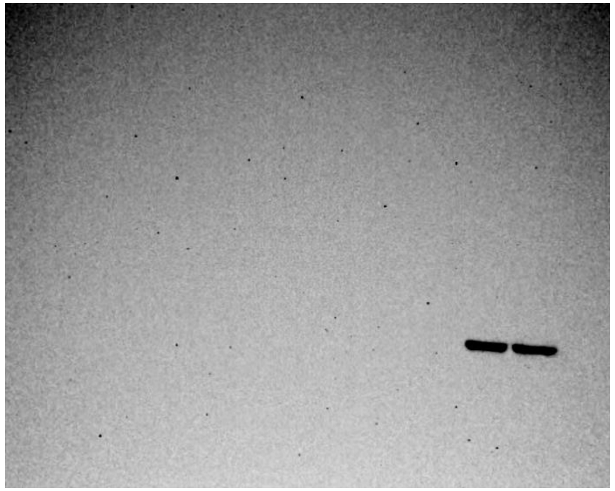

Figure 6J: WB results
Repeat 3

p53

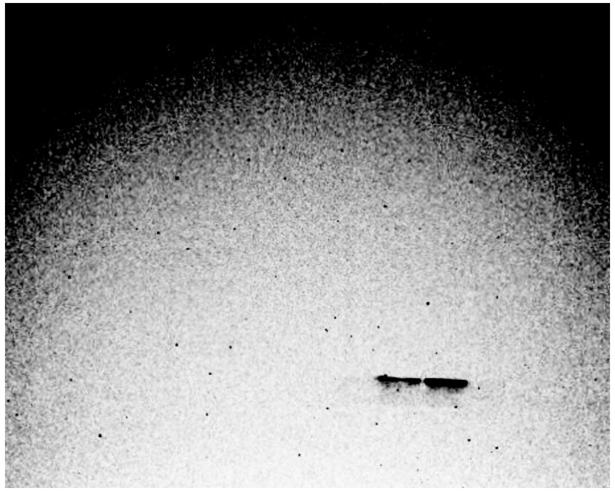

p21

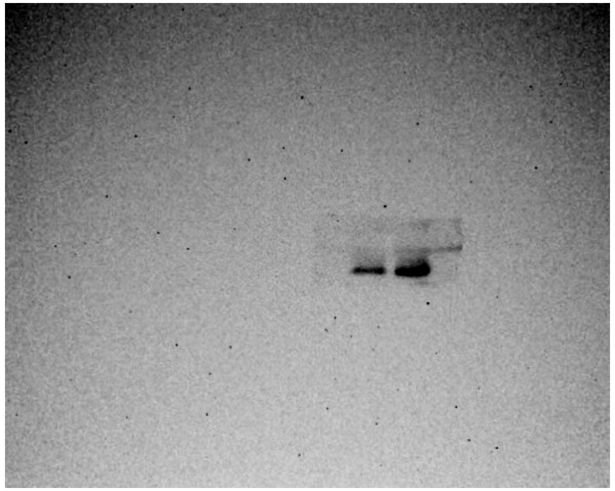

Actin

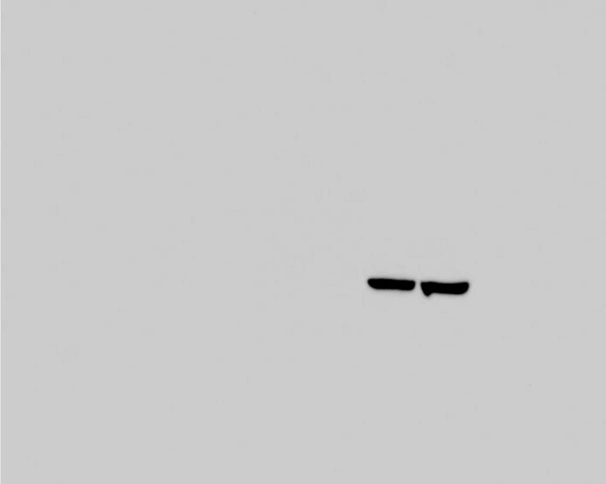

Figure 6K: WB results
Repeat 1

p53

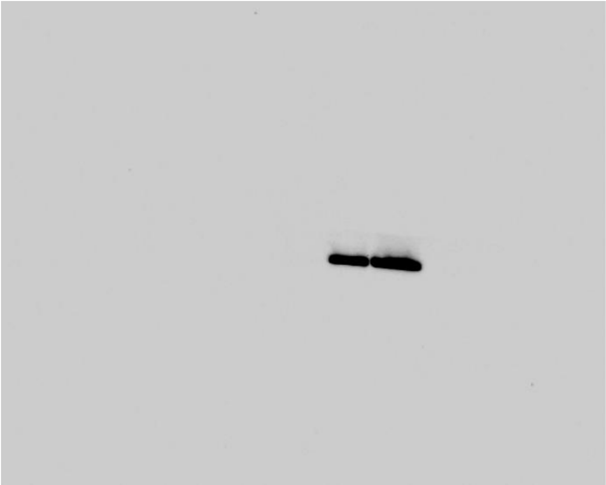

p21

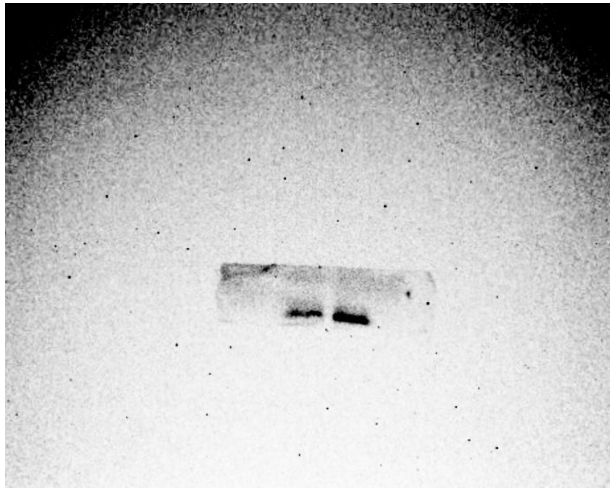

Actin

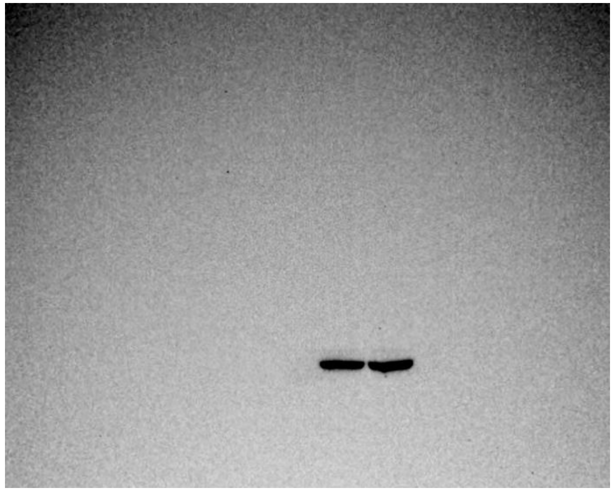

Figure 6K: WB results
Repeat 2

p53

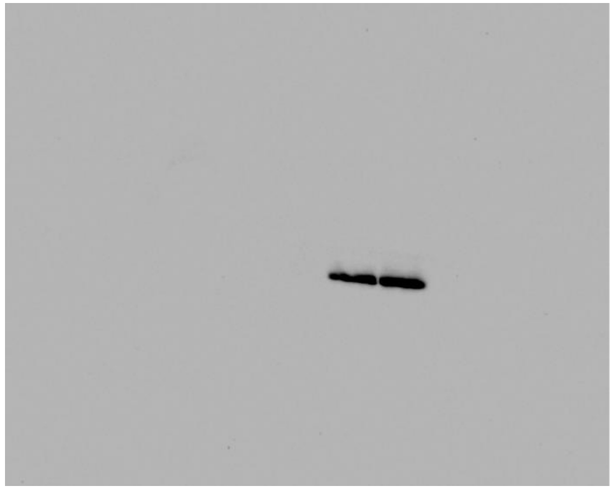

p21

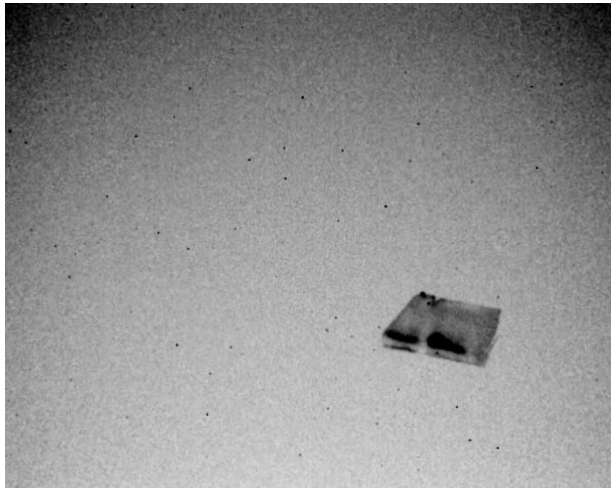

Actin

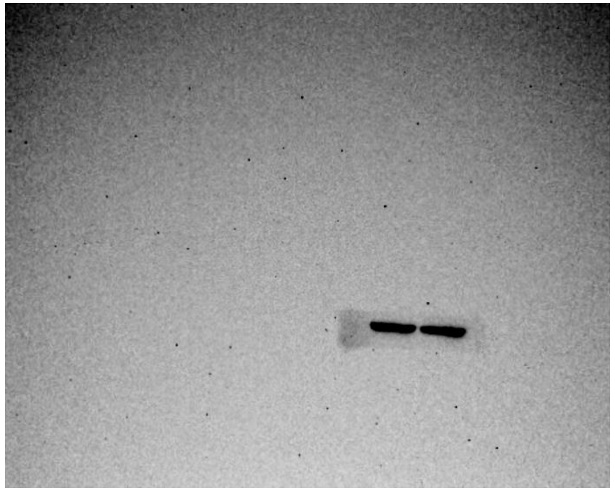

Figure 6K: WB results
Repeat 3

p53

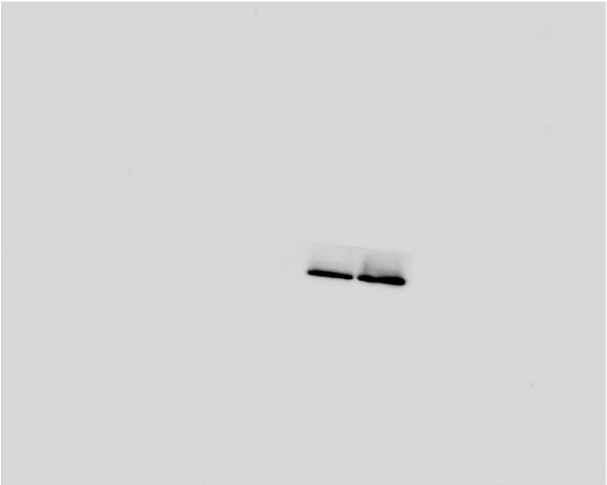

p21

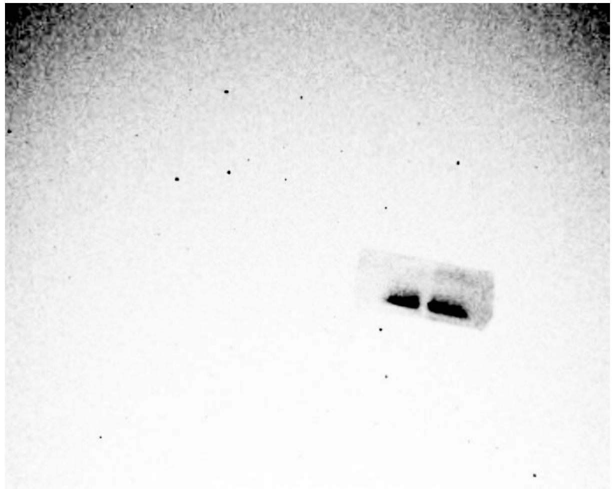

Actin

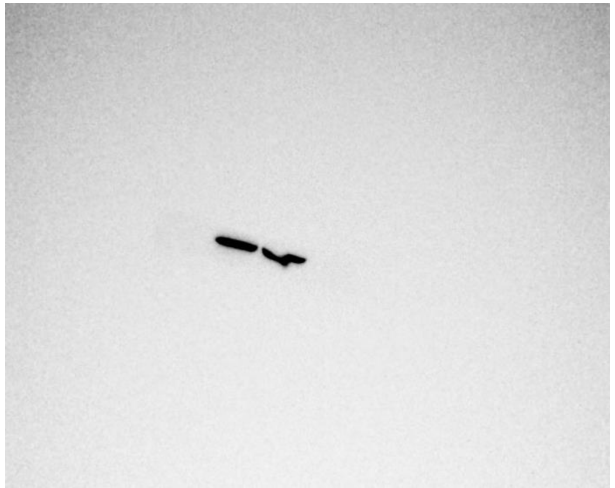

Supplementary Figure S2E: WB results
Repeat 1

IDO1

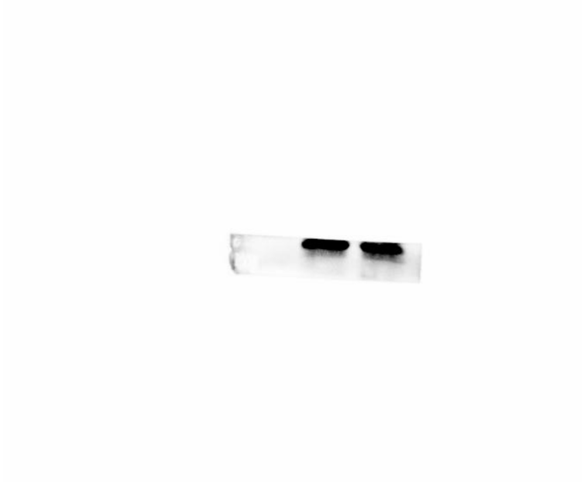

MDM2

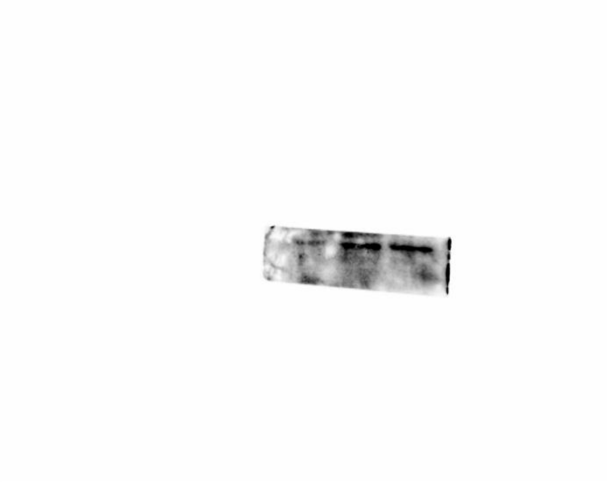

p53

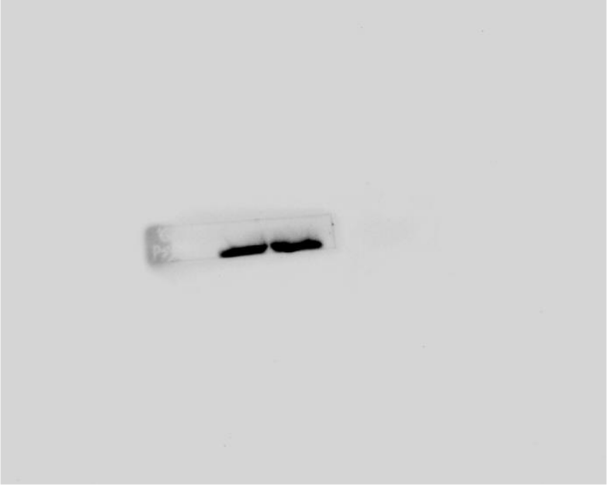

Actin

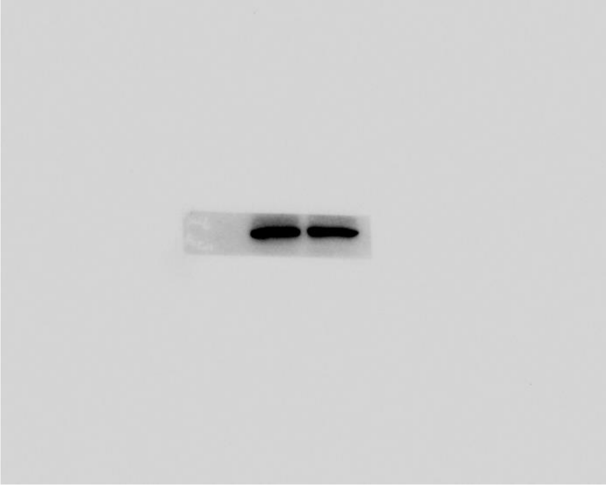

Supplementary Figure S2E: WB results
Repeat 2

IDO1

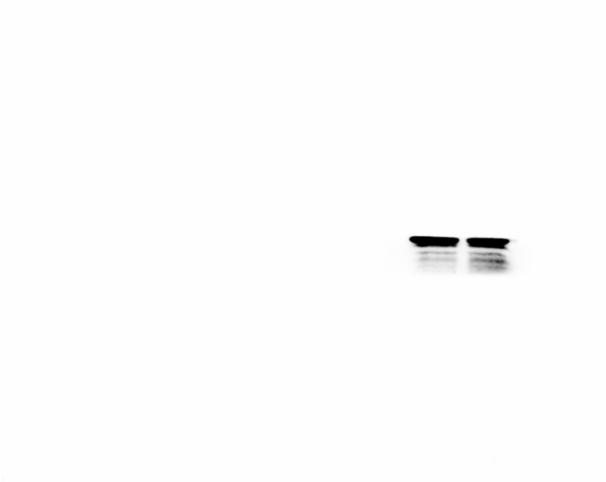

MDM2

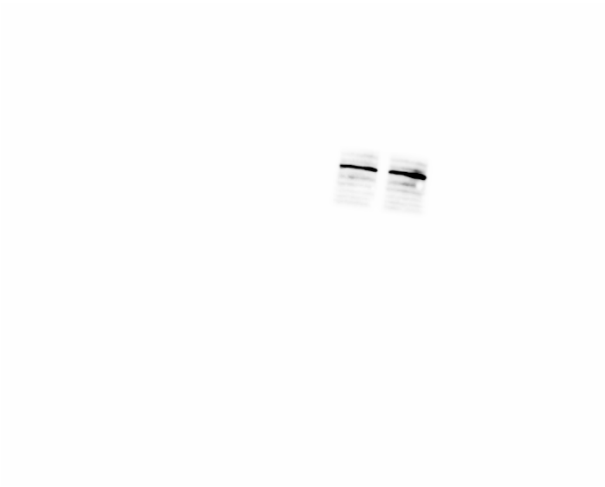

p53

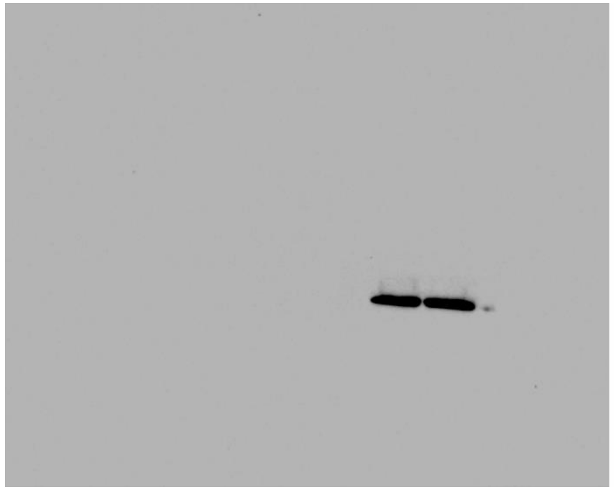

Actin

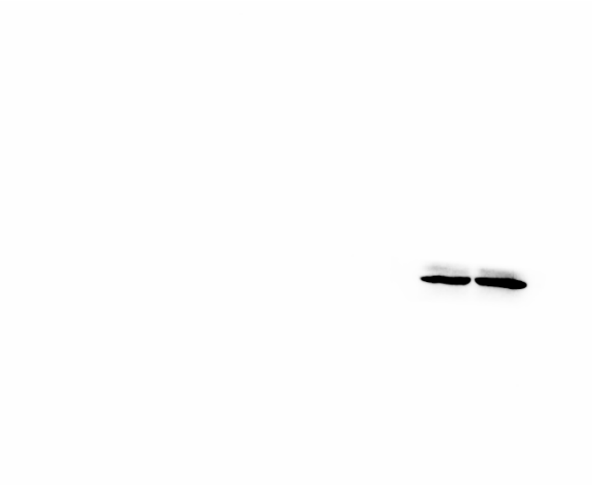

Supplementary Figure S2E: WB results
Repeat 3

IDO1 MDM2

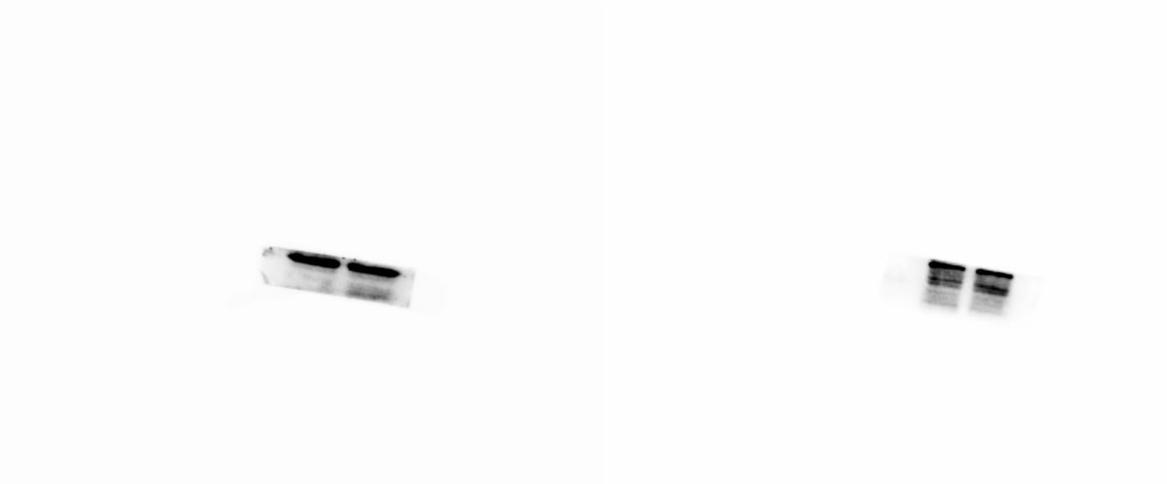

p53 Actin

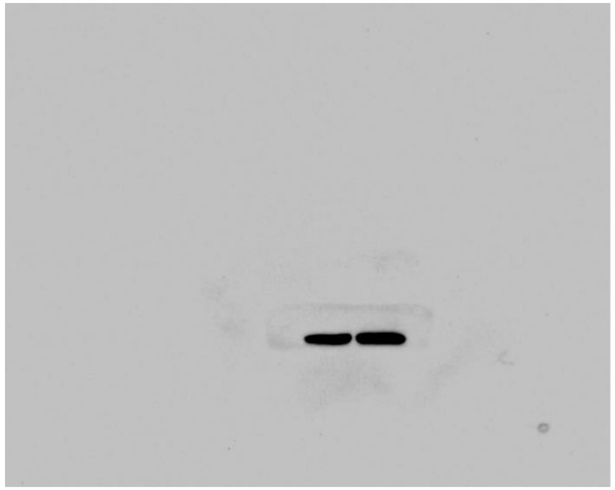

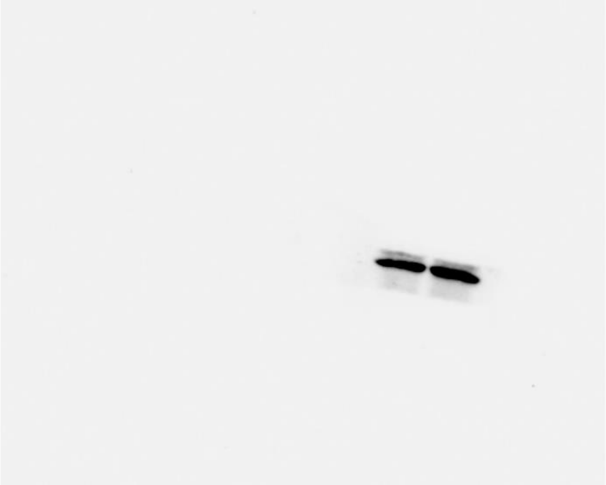

Supplementary Figure S2F: WB results

Repeat 1

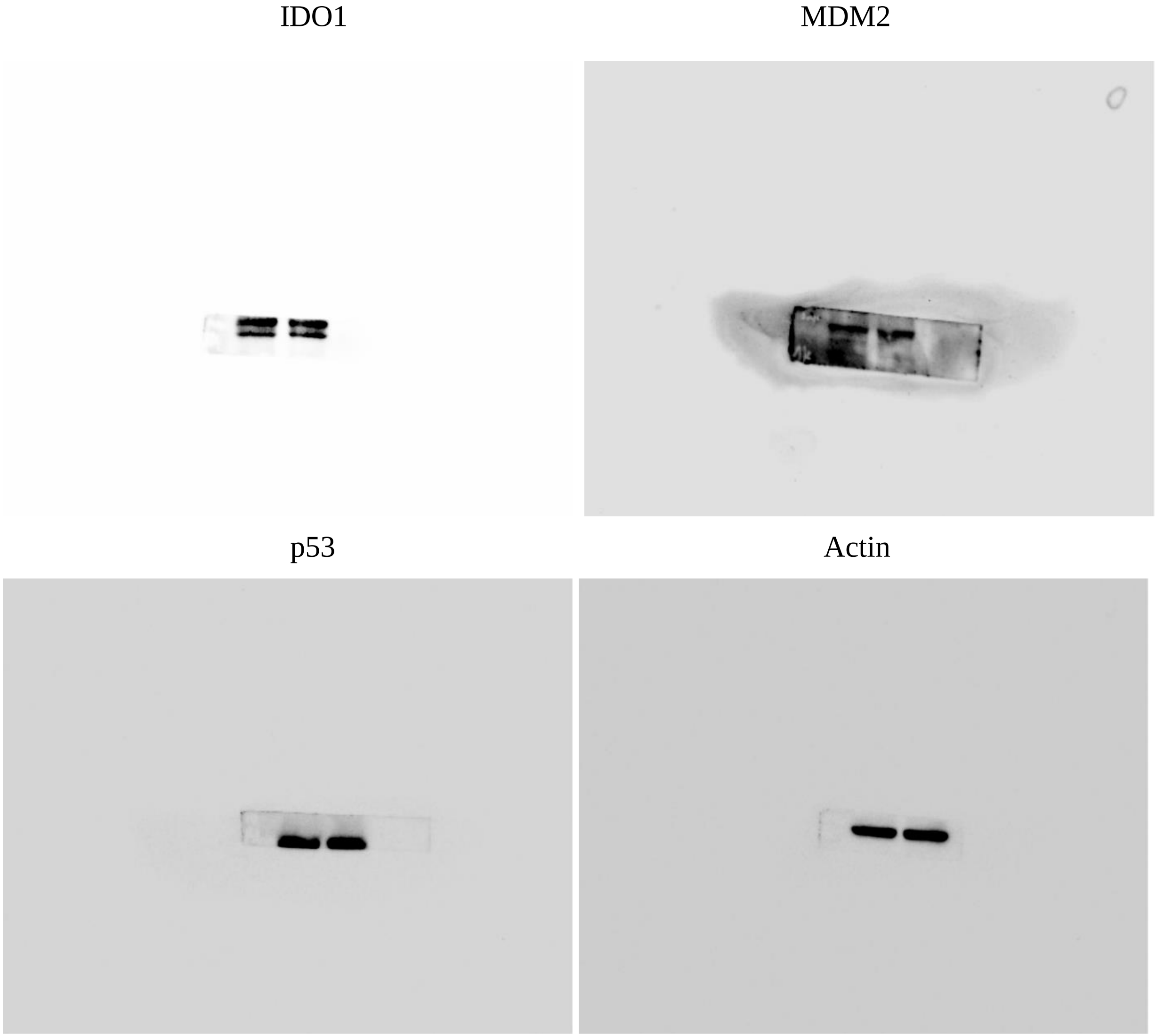

Supplementary Figure S2F: WB results
Repeat 2

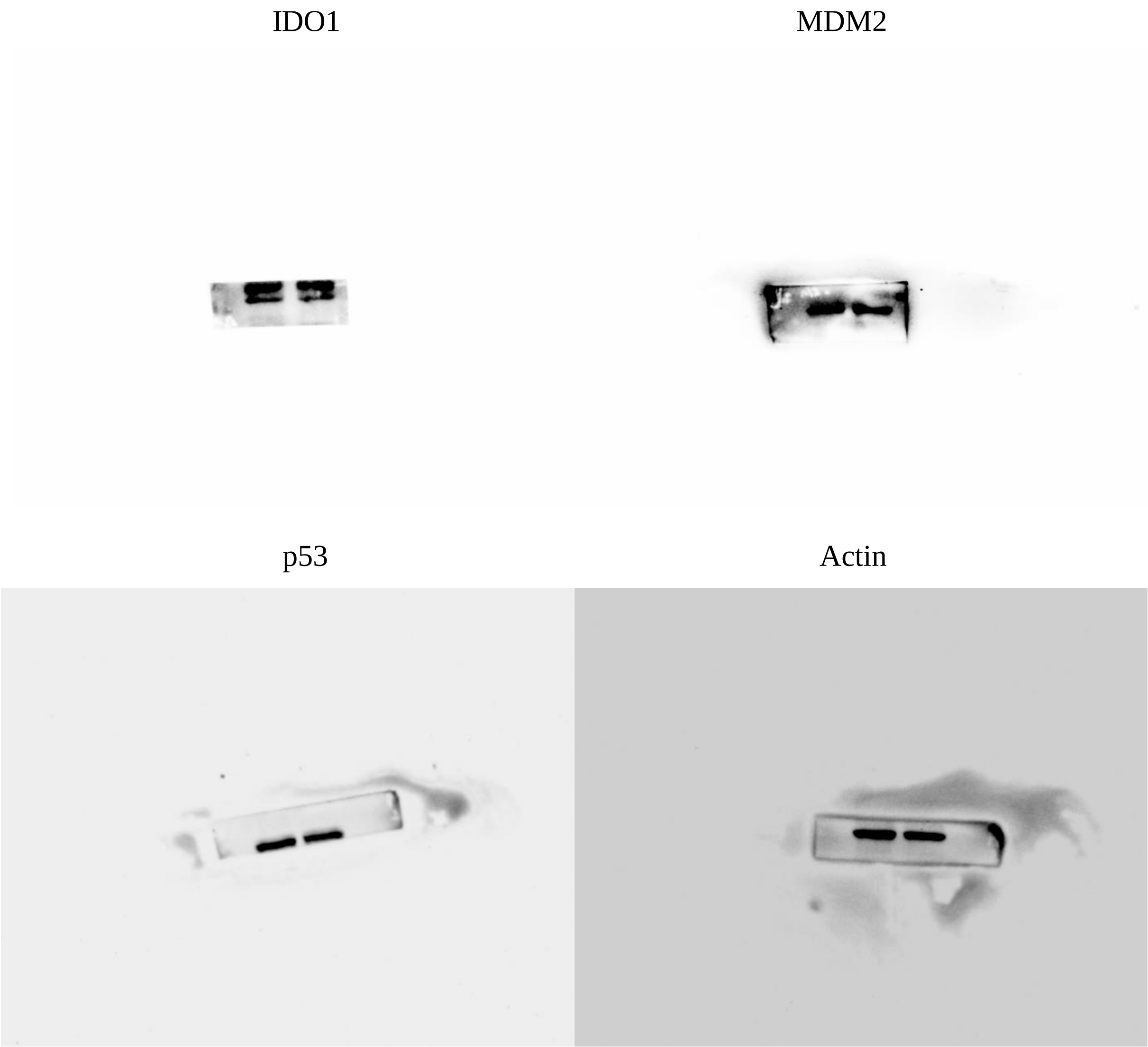

Supplementary Figure S2F: WB results
Repeat 3

IDO1

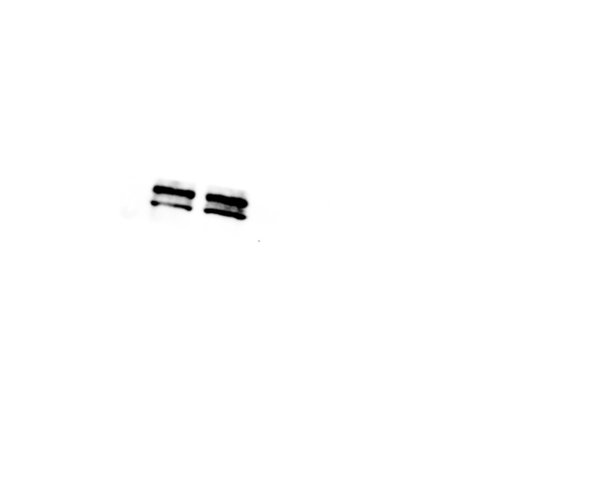

MDM2

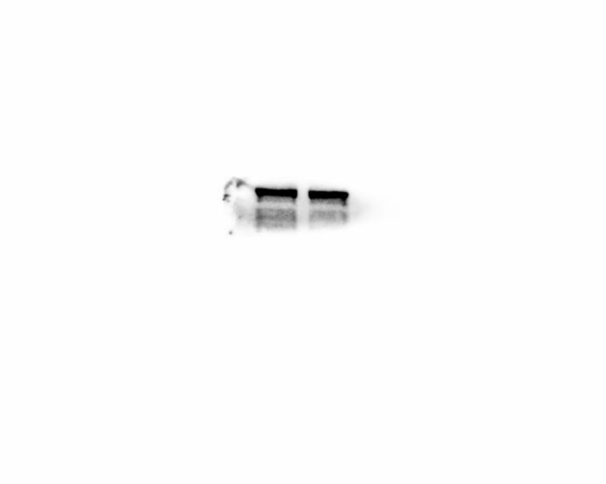

p53

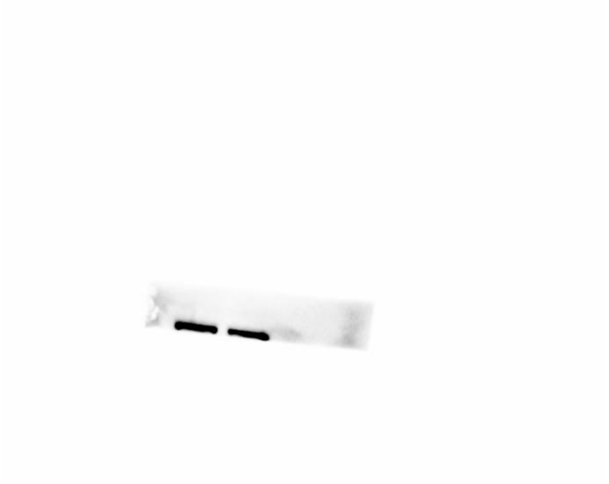

Actin

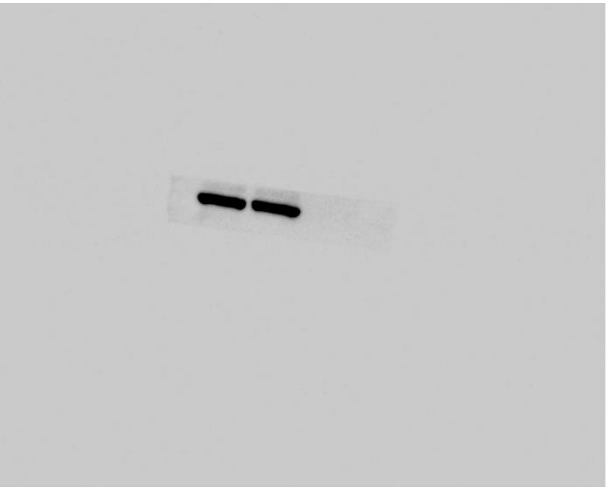

Supplementary Figure S2I: WB results
Repeat 1

BAX

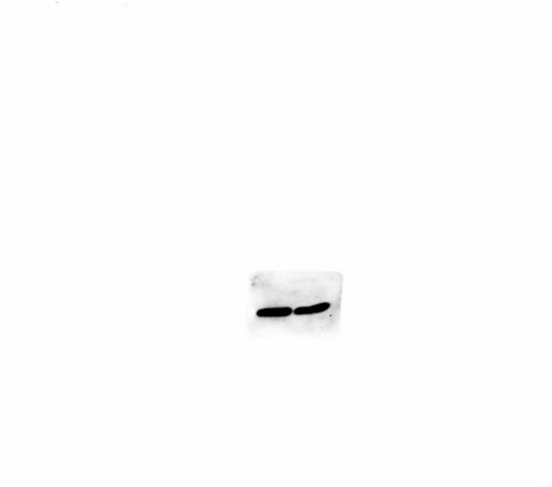

PUMA

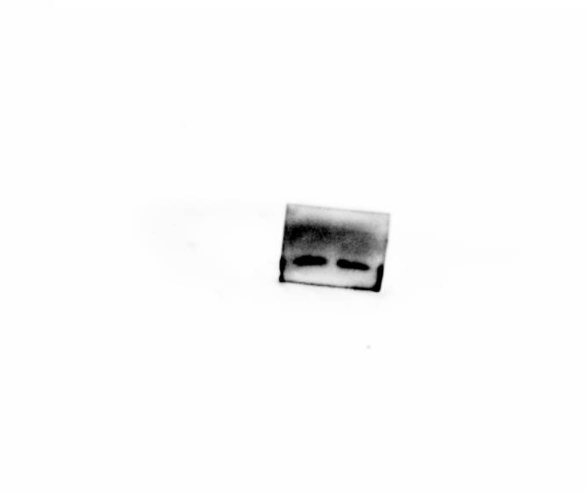

p21

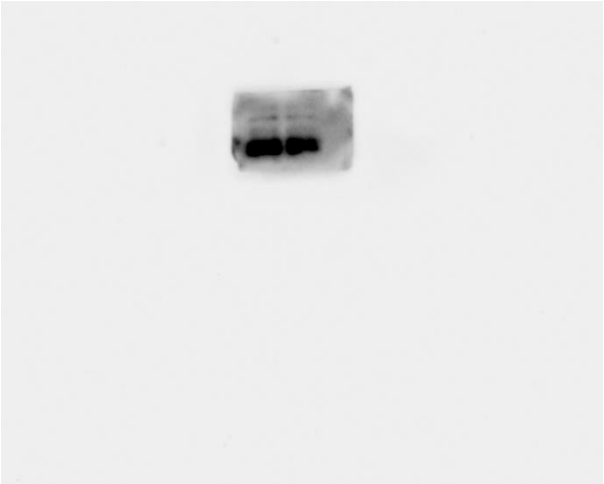

Actin

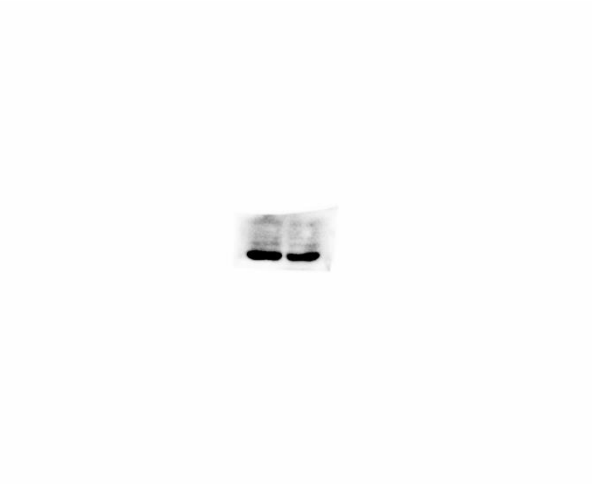

Supplementary Figure S2I: WB results
Repeat 2

BAX

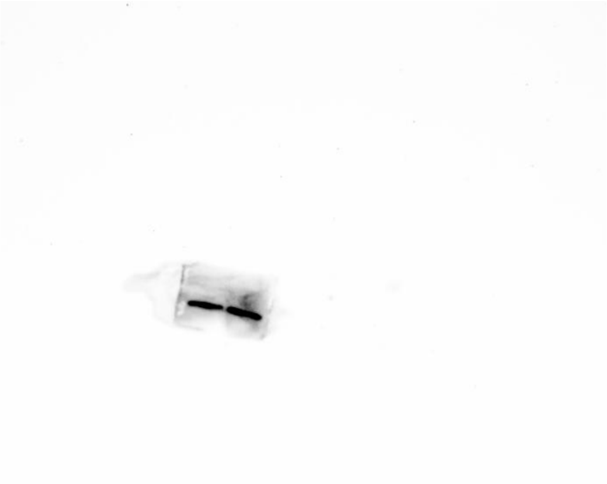

PUMA

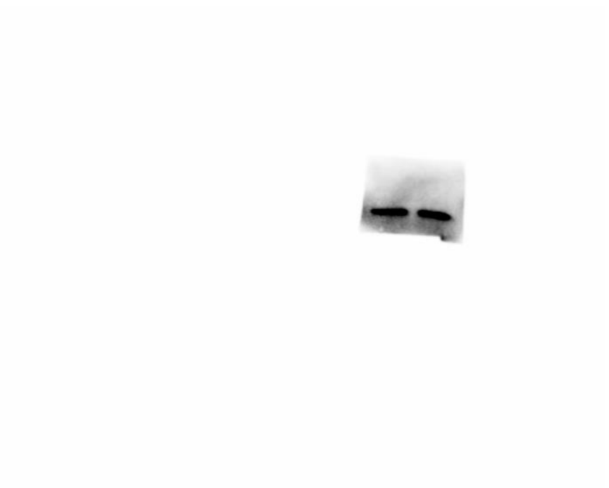

p21

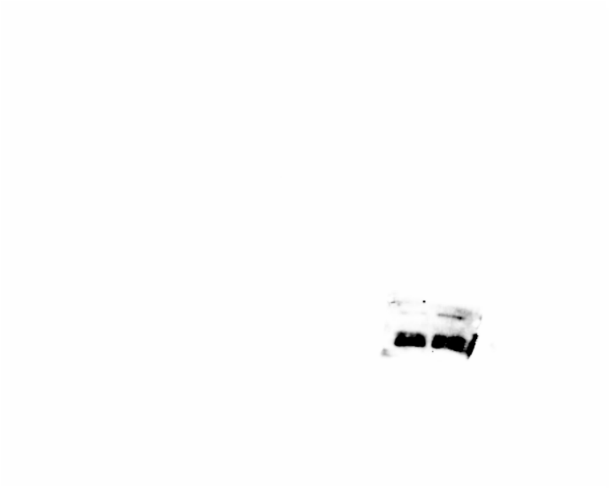

Actin

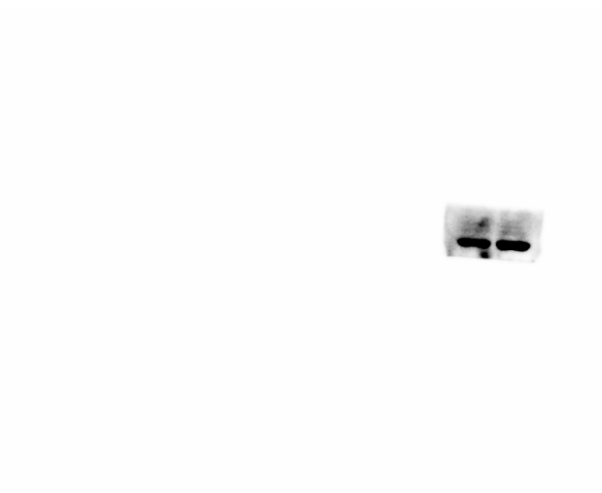

Supplementary Figure S2I: WB results
Repeat 3

BAX

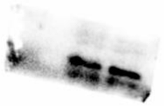

PUMA

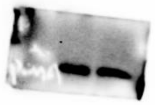

p21

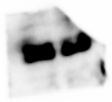

Actin

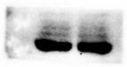

Supplementary Figure S2J: WB results
Repeat 1

BAX

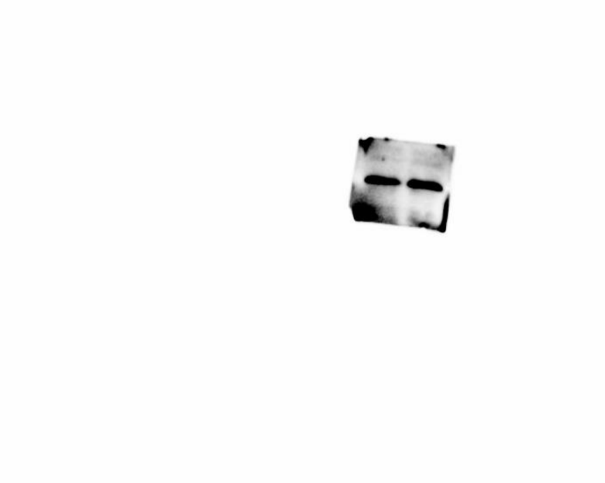

PUMA

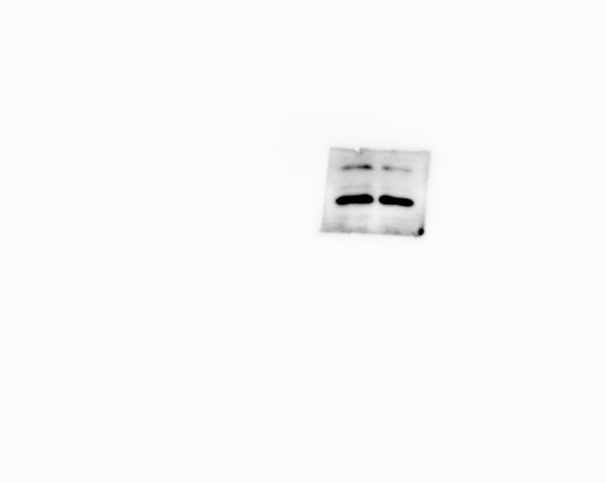

p21

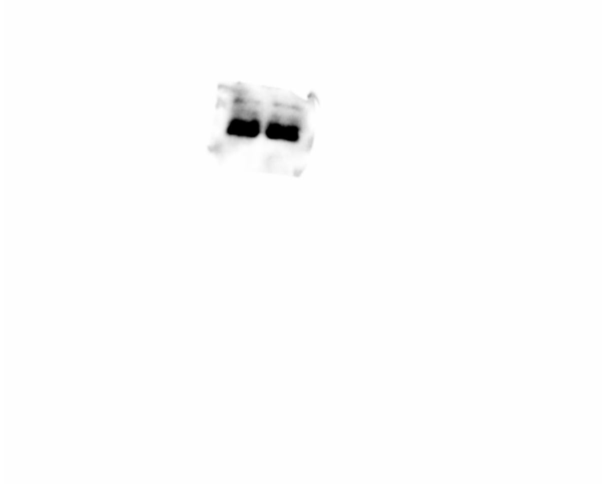

Actin

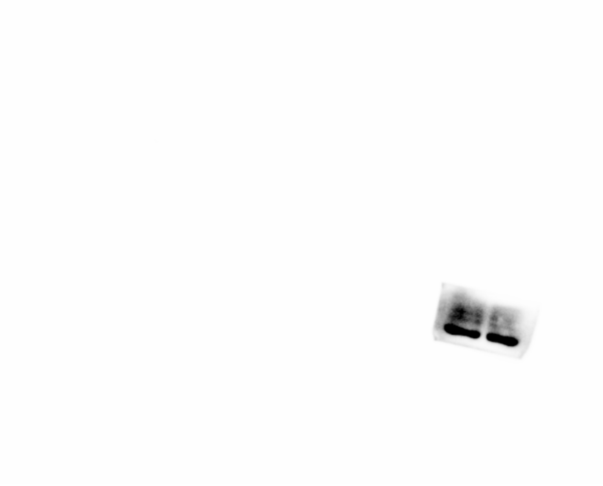

Supplementary Figure S2J: WB results
Repeat 2

BAX

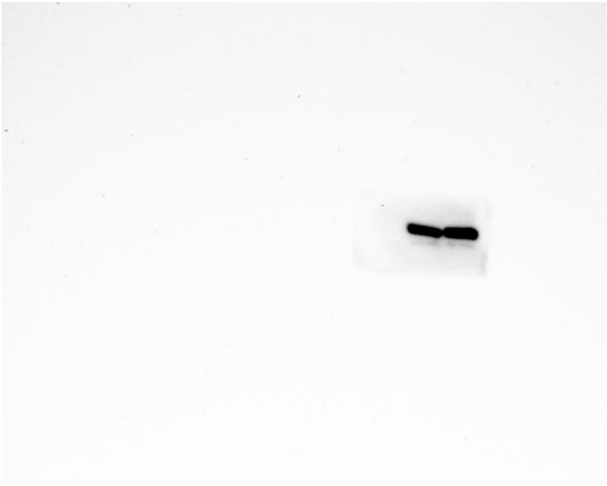

PUMA

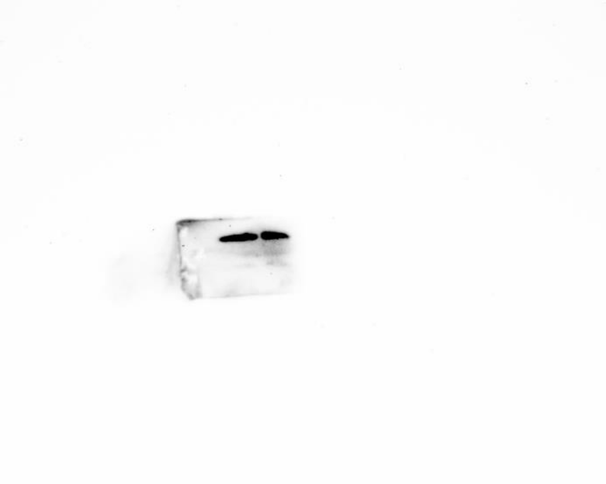

p21

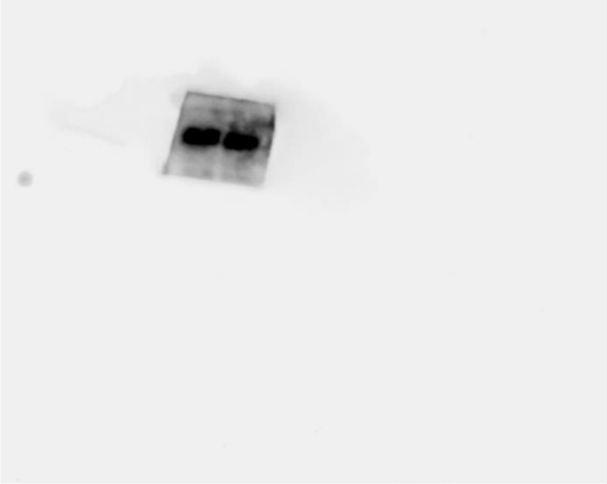

Actin

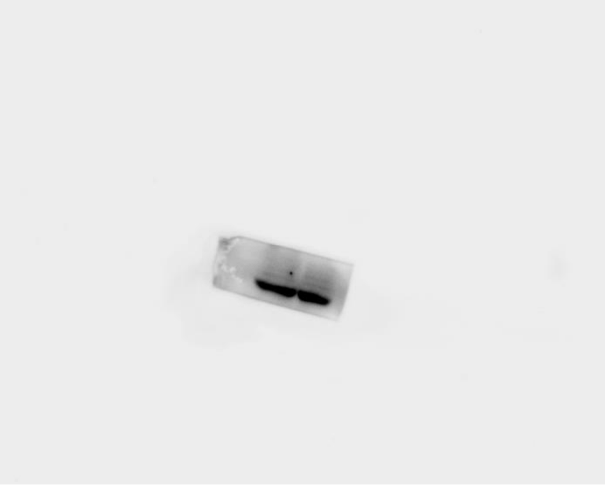

Supplementary Figure S2J: WB results
Repeat 3

BAX

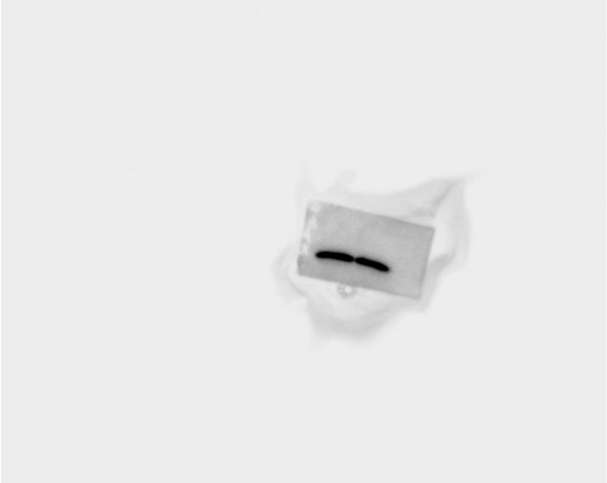

PUMA

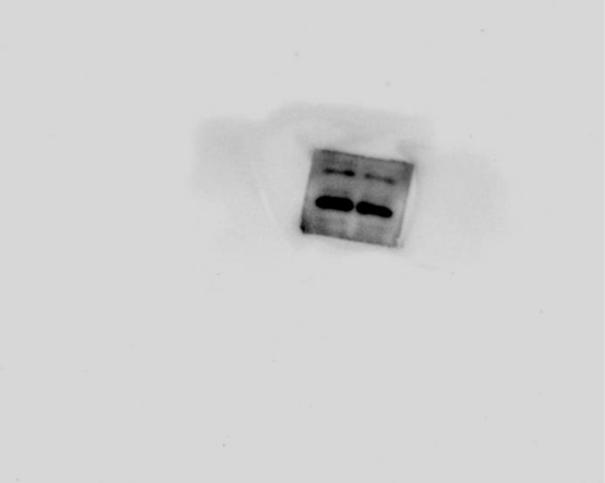

p21

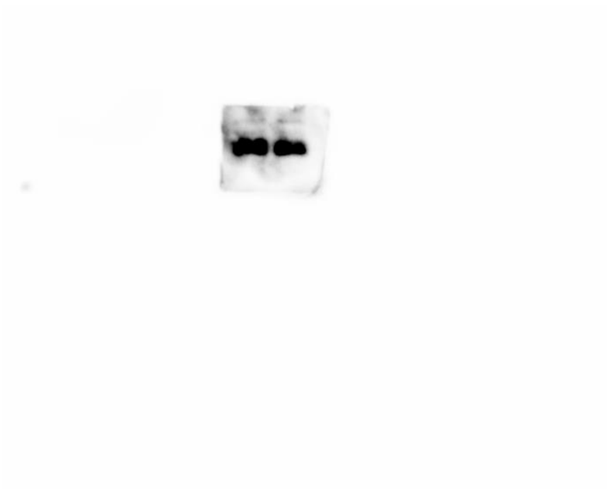

Actin

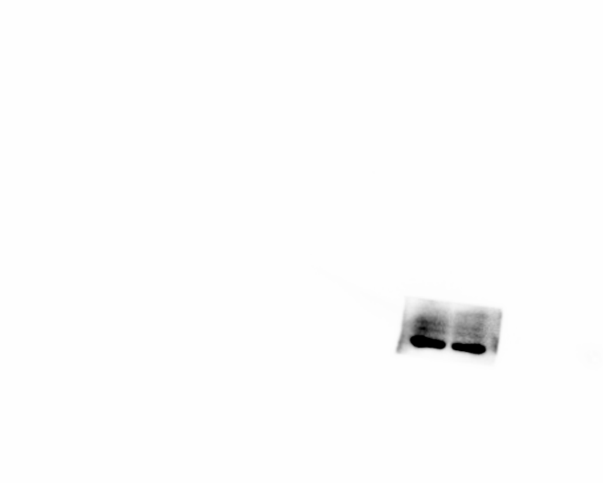

Supplement: Supplementary file 5 — Supplementary Figure S4 [file 41419_2022_5021_MOESM5_ESM.pdf]
