# Supplementary material for: IDO1 plays a tumor-promoting role via MDM2-mediated suppression of the p53 pathway in diffuse large B-cell lymphoma
Source: Cell Death Dis. 2022 Jun 27;13(6):572. doi: 10.1038/s41419-022-05021-2 (PMC9237101; doi:10.1038/s41419-022-05021-2)
Supplement: Supplementary file 6 — Supplementary Table S1 [file 41419_2022_5021_MOESM6_ESM.docx]

**Supplementary Table S1** The confusion matrix of GSE12195 and GSE56315

GSE12195

|  | Reference | |
| --- | --- | --- |
| Prediction | Normal | Tumor |
| Normal | 10 | 2 |
| Tumor | 0 | 71 |
| GSE56315 | | |
|  | Reference | |
| Prediction | Normal | Tumor |
| Normal | 33 | 0 |
| Tumor | 0 | 89 |
