# Supplementary material for: IDO1 plays a tumor-promoting role via MDM2-mediated suppression of the p53 pathway in diffuse large B-cell lymphoma
Source: Cell Death Dis. 2022 Jun 27;13(6):572. doi: 10.1038/s41419-022-05021-2 (PMC9237101; doi:10.1038/s41419-022-05021-2)
Supplement: Supplementary file 1 — co-author agreement file [file 41419_2022_5021_MOESM1_ESM.pdf]

From yizhuo zhang<zhangyzh@sysucc.org.cn>

Date 06/09/2022 21:03

To chengtao sun <mysuna@126.com>,
limz1<limz1@sysucc.org.cn>,
zhanglian2<zhanglian2@sysucc.org.cn>,
sunff<sunff@sysucc.org.cn>,
chenhm4<chenhm4@sysucc.org.cn>,
1013127057<1013127057@qq.com>,
lanyx<lanyx@sysucc.org.cn>,
zhangli11<zhangli11@sysucc.org.cn>,
lusy<lusy@sysucc.org.cn>,
zhujia<zhujia@sysucc.org.cn>,
huangjt<huangjt@sysucc.org.cn>,
wangjuan<wangjuan@sysucc.org.cn>,
huyang<huyang@sysucc.org.cn>,
fengyf<fengyf@sysucc.org.cn>

Subject Authorship Change Letter

Dear co-authors:

We are wishing to add Dr. Yingxia Lan as the seventh author of our article (manuscript number CDDIS-21-2748RRRR) entitled “IDO1 plays a tumor-promoting role via MDM2-mediated suppression of the p53 pathway in diffuse large B-cell lymphoma”, as described below (Authors: Chengtao Sun#, Mengzhen Li#, Lian Zhang#, Feifei Sun, Huimou Chen, Yanjie Xu, Yingxia Lan, Li Zhang, Suying Lu, Jia Zhu, Junting Huang, Juan Wang, Yang Hu, Yanfen Feng, and Yizhuo Zhang*). Yingxia Lan contributed much in the revised version of our manuscript. She did a great job for the in vivo study, GEO data analysis, further information on the subtypes of DLBCL, ELISA, precision recall curves (PRC) and receiver operating characteristic (ROC) analysis, as listed in revised Figure 1 D-G, Figure 2 F, Figure 3 H-I, Supplementary Figure S3, and Supplementary Table S1.

Please confirm whether you agree to the change in the authorship of our article.

Sincerely,

Yizhuo Zhang

The replies of Co-authors are listed as below:

Re: Authorship Change Letter
发件人：Chengtao Sun <mysuna@126.com>
时　间：2022 年 6 月 9 日(星期四) 凌晨 4:26
收件人：Yizhuo Zhang <zhangyzh@sysucc.org.cn>
Dear professor Zhang,
I agree the Authorship Change in our manuscript.

RE: Authorship Change Letter
发件人：Mengzhen Li <limz1@sysucc.org.cn>
时　间：2022 年 6 月 9 日(星期四) 凌晨 4:42
收件人：Yizhuo Zhang <zhangyzh@sysucc.org.cn>
Yes， I agree to this addition of the author.

Mengzhen Li
09/06/2022

RE: Authorship Change Letter
发件人：Lian Zhang <zhanglian2@sysucc.org.cn>
时　间：2022 年 6 月 9 日(星期四) 凌晨 4:24
收件人：Yizhuo Zhang <zhangyzh@sysucc.org.cn>
Agreed. Dr.Lan did good job and made great contributions on data analysis during manuscript revision.

Best,
Lian

Re:Authorship Change Letter
发件人：Feifei Sun <sunff@sysucc.org.cn>
时　间：2022 年 6 月 10 日(星期五) 下午 4:08
收件人：Yizhuo Zhang <zhangyzh@sysucc.org.cn>
I agree to the change in the authorship of our article.

RE：Authorship Change Letter
发件人：Huimou Chen <chenhm4@sysucc.org.cn>
时　间：2022 年 6 月 9 日(星期四) 下午 2:19
收件人：Yizhuo Zhang <zhangyzh@sysucc.org.cn>
Yes, I agree to the change in the authorship of our article.
Thank you.

Sincerely,

Huimou Chen

RE: Authorship Change Letter
发件人：Yanjie Xu <1013127057@qq.com>
时　间：2022 年 6 月 9 日(星期四) 凌晨 5:26
收件人：Yizhuo Zhang <zhangyzh@sysucc.org.cn>
Dear Dr. Yizhuo Zhang

I agree to add Dr. Yingxia Lan as the seventh author of our article (manuscript number CDDIS-21-2748RRRR) entitled “IDO1 plays a tumor-promoting role via MDM2-mediated suppression of the p53 pathway in diffuse large B-cell lymphoma”.

Sincerely,

Yanjie Xu

RE:Authorship Change Letter
发件人：Yingxia Lan <lanyx@sysucc.org.cn>
时　间：2022 年 6 月 9 日(星期四) 凌晨 4:18
收件人：Yizhuo Zhang <zhangyzh@sysucc.org.cn>
I agree to the change in the authorship of our article.

Re:Authorship Change Letter
发件人：Li Zhang <zhangli11@sysucc.org.cn>
时　　间：2022 年 6 月 9 日(星期四) 下午 3:23
收件人：Yizhuo Zhang <zhangyzh@sysucc.org.cn>

yes, I agree

Re:Authorship Change Letter
发件人：Suying Lu <lusy@sysucc.org.cn>
时　　间：2022 年 6 月 9 日(星期四) 中午 1:38
收件人：Yizhuo Zhang <zhangyzh@sysucc.org.cn>
Yes, I agree to the change in the authorship of our article.
Thank you.

Sincerely,

Suying Lu

Re:Authorship Change Letter
发件人：Jia Zhu <zhujia@sysucc.org.cn>
时　　间：2022 年 6 月 9 日(星期四) 下午 2:53
收件人：Yizhuo Zhang <zhangyzh@sysucc.org.cn>
Dear Professor Zhang:

I totally agree to add Dr. Yingxia Lan as the seventh author of our article (manuscript number CDDIS-21-2748RRRR) entitled “IDO1 plays a tumor-promoting role via MDM2-mediated suppression of the p53 pathway in diffuse large B-cell lymphoma”.

Sincerely,
Jia Zhu

Re:Authorship Change Letter
发件人：Junting Huang<huangjt@sysucc.org.cn>
时　　间：2022 年 6 月 9 日(星期四) 晚上 11:17
收件人：Yizhuo Zhang <zhangyzh@sysucc.org.cn>
I agree adding Dr. Yingxia Lan as the seventh author.

Re:Authorship Change Letter
发件人：Juan Wang <wangjuan@sysucc.org.cn>
时　　间：2022 年 6 月 9 日(星期四) 凌晨 4:21
收件人：Yizhuo Zhang <zhangyzh@sysucc.org.cn>
Dear Prof. Zhang:

I agree with the change of Dr. Yingxia Lan as the seventh author of the article (manuscript number CDDIS-21-2748RRRR) entitled “IDO1 plays a tumor-promoting role via MDM2-mediated suppression of the p53 pathway in diffuse large B-cell lymphoma”.

Sincerely,

Juan Wang

Re: Authorship Change Letter
发件人：Yang Hu <huyang@sysucc.org.cn>
时　　间：2022 年 6 月 9 日(星期四) 凌晨 4:21
收件人：Yizhuo Zhang <zhangyzh@sysucc.org.cn>
I agree to add Dr. Yingxia Lan as the seventh author of our article (manuscript number CDDIS-21-2748RRRR) entitled “IDO1 plays a tumor-promoting role via MDM2-mediated suppression of the p53 pathway in diffuse large B-cell lymphoma”.
Thanks.

Re: Authorship Change Letter
发件人：Yanfen Feng <fengyf@sysucc.org.cn>
时　　间：2022年6月9日(星期四) 凌晨4:14
收件人：Yizhuo Zhang <zhangyzh@sysucc.org.cn>
Dear Prof. Zhang
　I agree the change in the authorship of our article.
